# Cooperation between somatic mutation and germline-encoded residues enables antibody recognition of HIV-1 envelope glycans

**DOI:** 10.1371/journal.ppat.1008165

**Published:** 2019-12-16

**Authors:** Nelson R. Wu, Nathan I. Nicely, Esther M. Lee, Rachel K. Reed, Brian E. Watts, Fangping Cai, William E. Walkowicz, Baptiste Aussedat, Julia A. Jones, Amanda Eaton, Ashley M. Trama, S. Munir Alam, David C. Montefiori, Barton F. Haynes, Kevin O. Saunders

**Affiliations:** 1 Department of Medicine, Duke University Medical Center, Durham, North Carolina, United States of America; 2 Duke Human Vaccine Institute, Duke University Medical Center, Durham, North Carolina, United States of America; 3 Department of Chemical Biology, Memorial Sloan Kettering Cancer Center, New York, New York, United States of America; 4 Department of Surgery, Duke University Medical Center, Durham, North Carolina, United States of America; 5 Department of Biomedical Engineering, Duke University Medical Center, Durham, North Carolina, United States of America; 6 Department of Immunology, Duke University Medical Center, Durham, North Carolina, United States of America; 7 Department of Molecular Genetics and Microbiology, Duke University Medical Center, Durham, North Carolina, United States of America; Vaccine Research Center, UNITED STATES

## Abstract

Viral glycoproteins are a primary target for host antibody responses. However, glycans on viral glycoproteins can hinder antibody recognition since they are self glycans derived from the host biosynthesis pathway. During natural HIV-1 infection, neutralizing antibodies are made against glycans on HIV-1 envelope glycoprotein (Env). However, such antibodies are rarely elicited with vaccination. Previously, the vaccine-induced, macaque antibody DH501 was isolated and shown to bind to high mannose glycans on HIV-1 Env. Understanding how DH501 underwent affinity maturation to recognize glycans could inform vaccine induction of HIV-1 glycan antibodies. Here, we show that DH501 Env glycan reactivity is mediated by both germline-encoded residues that contact glycans, and somatic mutations that increase antibody paratope flexibility. Only somatic mutations in the heavy chain were required for glycan reactivity. The paratope conformation was fragile as single mutations within the immunoglobulin fold or complementarity determining regions were sufficient for eliminating antibody function. Taken together, the initial germline V_H_DJ_H_ rearrangement generated contact residues capable of binding glycans, and somatic mutations were required to form a flexible paratope with a cavity conducive to HIV-1 envelope glycan binding. The requirement for the presence of most somatic mutations across the heavy chain variable region provides one explanation for the difficulty in inducing anti-Env glycan antibodies with HIV-1 Env vaccination.

## Author summary

The viral pathogen HIV-1 uses sugar molecules, called glycans, from the host to densely cover its envelope protein. Most broadly neutralizing HIV-1 antibodies interact with glycans on the HIV-1 envelope protein. For this reason, the vaccine induction of anti-HIV-1 glycan antibodies is a principal goal. Since vaccine-induced anti-HIV-1 glycan antibodies are rare, it has not been determined how antibodies develop during vaccination to recognize HIV-1 glycans. Here, we elucidated the amino acids required for a primate antibody induced by HIV-1 vaccination to interact with HIV envelope glycans. Genetic and functional analyses showed the putative antibody germline nucleotide sequence encoded amino acids that were required for glycan reactivity. Somatic mutation also introduced critical amino acids that were required for glycan recognition. Unusually, the somatic mutations were not required in order to form direct contacts with antigen, but instead functioned to improve antibody flexibility and to form its glycan binding site. These results define the molecular development of a vaccine-induced HIV-1 glycan antibody, providing insight into why vaccines rarely elicit antibodies against the glycans on the HIV-1 outer coat protein.

## Introduction

For many enveloped viruses the proteins on their surfaces are glycosylated by host enzymes during protein folding and transport from the endoplasmic reticulum and Golgi apparatus [1, 2]. The glycans on viral envelope proteins are critical for virus infectivity [3], as removal of certain glycans can reduce envelope incorporation into virions and envelope binding to its host cell receptors [3, 4]. Additionally, glycans on viral envelope glycoproteins provide a shield against host immune recognition [4–8], since the host antibody repertoire has limited antibodies specific for host glycans [9, 10]. Thus, envelope glycosylation is beneficial for virus infectivity, and it creates a formidable challenge for the development of humoral immunity against viral envelope glycoproteins.

Human immunodeficiency virus subtype 1 (HIV-1) provides a model pathogen to study glycan-specific antibody responses since its surface glycoprotein is one the most heavily glycosylated proteins known [10–12]. On average the HIV-1 envelope (Env) glycoprotein contains 22 different N-linked glycosylation sites [11, 13, 14]. These sites can vary with respect to glycan occupancy and composition [8, 15–17], which creates substantial heterogeneity among HIV-1 Env molecules. Moreover, the amino acid sequence of HIV-1 Env mutates to relocate glycosylation sites, contributing to Env diversity and immune escape variants [8].

Antibodies that bind to HIV-1 Env glycans arise during natural infection [18–23]. Crystal structures have revealed that these antibodies bind to glycan as well as peptide [24–27]. One exception is the neutralizing antibody 2G12, which binds only to glycans within the high mannose patch on Env [28, 29]. HIV-1 glycan-reactive antibodies are attractive vaccine targets. Individual glycan-reactive antibodies or combinations of two of these antibodies can neutralize 80% or 90% of diverse, circulating HIV-1 isolates, respectively [20, 22]. Additionally, passive transfer of glycan-reactive HIV-1 antibodies has shown them to be protective in macaque models of mucosal HIV-1 infection [5, 6, 30, 31].

In contrast to natural HIV-1 infection, vaccine induction of glycan-reactive HIV-1 antibodies has been rarely achieved [32–36]. In a previous HIV-1 vaccine study, we isolated a monoclonal antibody, DH501, from a group M consensus Env-immunized rhesus macaque [37]. DH501 typified a new type of HIV-1 glycan antibody that required the N301 glycan on Env for neutralization and bound directly to high mannose glycans [37]. High mannose glycans are abundant on HIV-1 Env, but not normally found on human proteins [17, 38–40]. The crystal structure of DH501 bound to Man_9_GlcNAc_2_ showed that it bound to the terminal mannose residues on Man_9_GlcNAc_2_ with a deep cavity formed by its heavy chain complementarity determining regions [CDRs; 37]. The somatic mutations required for DH501 glycan binding activity are unknown, but appear to be important since its inferred germline precursor, the unmutated common ancestor of DH501 (DH501 UCA), lacked glycan reactivity and Env binding [37]. The disparate glycan reactivities of the DH501 UCA and DH501 provides an opportunity to delineate the role of specific somatic mutations in antibody affinity maturation to HIV-1 envelope glycans.

Here, we combined structural and site-specific mutational analyses to determine the amino acids responsible for glycan reactivity in the DH501 antibody. The structural evolution of the glycan-binding cavity was determined by solving the crystal structure of the DH501 UCA antibody. Reversion of DH501 somatic mutations to the unmutated common ancestor (UCA) sequence showed that the glycan-binding cavity required simultaneous mutation of framework regions (FWRs) and CDRs. These somatic mutations did not produce new contacts with antigen, but instead generated a flexible paratope conducive for antigen recognition. Additionally, germline-encoded amino acids were functionally important as they formed required contacts with glycan. This study shows that vaccines elicit glycan-reactive antibodies by somatically mutating CDRs and FWRs to increase the flexibility of variable regions that possess putative germline amino acids capable of binding glycans.

## Results

### Somatic mutation of the DH501 heavy chain is sufficient for glycan recognition

**Fig 1:**
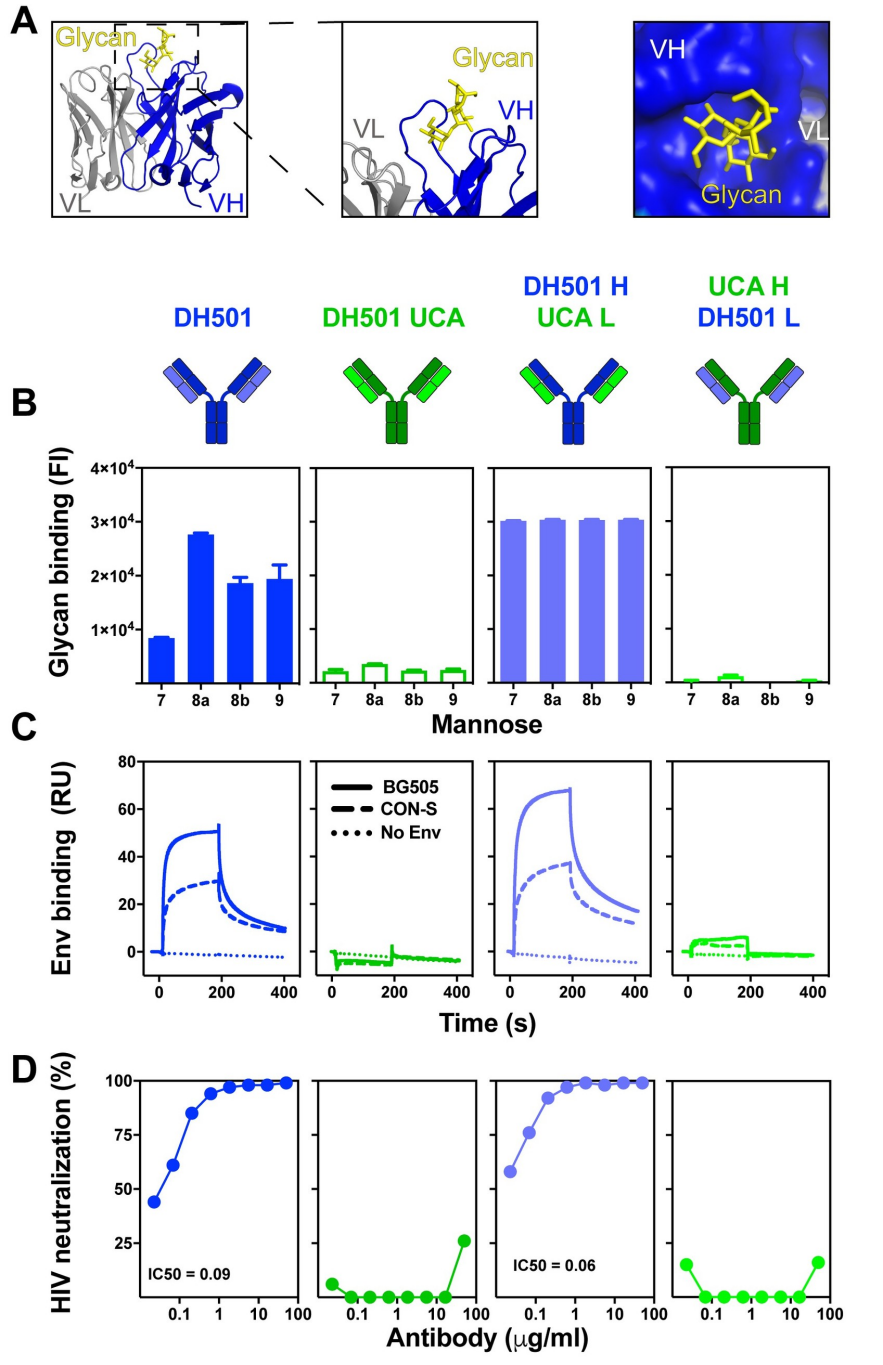
DH501 heavy chain somatic mutations, but not light chain mutations, are required for glycan reactivity and trimeric HIV-1 envelope recognition. **(A)** Crystal structure of DH501 Fab (PDB: 5T4Z) bound to the terminal mannose residues of the D2 arm of Man_9_GlcNAc_2_. The Fab is shown in cartoon form with the Man_9_GlcNAc_2_ shown by stick structures. Magnified images in the middle and right show mannose residues contacting only heavy chain amino acids (blue). Image created in Pymol [41]. **(B)** Antibody binding to Man_7_GlcNAc_2_ D1, Man_8_GlcNAc_2_ D1D3, Man_8_GlcNAc_2_ D1D2, Man_9_GlcNAc_2_, which are denoted as 7, 8a, 8b, and 9 respectively. Throughout **Fig 1** functional data is shown for the antibody listed at the top of the column. Antibody functions were assessed for DH501 with somatic mutations in both the heavy and light chain (DH501), with no somatic mutations (DH501 UCA), with somatic mutations only in the heavy chain (DH501 H/ UCA L), and with somatic mutations only in the light chain (UCA H/DH501 L). The mean and standard error are shown for independent triplicate experiments. DH501 H/ UCA L reached the upper limit of detection (fluorescence intensity of 31,000). Positive binding based on negative control antibody binding is shown as a filled bar. Open bars indicate negative binding values. Positivity thresholds for 7, 8a, 8b, and 9 are 0.6x10^4^, 0.90x10^4^, 0.67x10^4^, 0.64x10^4^ respectively. **(C)** Binding of various DH501 IgG variants to soluble, cleaved HIV-1 CON-S and BG505 SOSIP gp140 envelope. Dotted lines show binding to blank flow cells as a negative control. Values are representative of two independent experiments. **(D)** *In vitro* HIV-1 pseudovirus neutralization of kifunensine-treated (Man_9_GlcNAc_2_-enriched) JR-FL in the TZM-bl neutralization assay. Neutralization titers (IC50 in μg/mL) are shown for antibodies that neutralized virus replication 50% or greater.

In the crystal structure of DH501 Fab bound to free Man_9_GlcNAc_2_, we found that the heavy chain CDRs formed a glycan binding cavity into which the terminal glycans of the D2 arm of the mannose inserted [Fig 1A; 37]. The crystal structure showed that the light chain did not contact the glycan directly, and thus the requirement of somatic mutation in the light chain was unclear (**Fig 1A**). To determine the role of light chain somatic mutation in glycan binding, we reverted the somatic mutations in the variable region of the light chain (V_L_) back to their inferred UCA sequence, creating a DH501 H/UCA L chimeric antibody. For comparison, we also reverted the heavy chain variable region (V_H_) mutations to UCA sequence to create the UCA H/DH501 L chimeric antibody. We tested the binding of all four antibodies to Man_7_GlcNAc_2_, Man_8_GlcNAc_2_, and Man_9_GlcNAc_2_ glycans (**Fig 1B** and **S1 Fig**). Both DH501 and DH501 H/UCA L bound to high mannose glycans (**Fig 1B**), with the DH501 H/UCA L exhibiting higher binding magnitudes. Reversion of the V_H_ mutations (UCA H/DH501 L) or reverting all mutations (UCA) in the antibody eliminated glycan binding (**Fig 1B**).

Next we investigated whether the DH501 variants that had lost glycan reactivity were capable of binding to trimeric Env in surface plasmon resonance and virus neutralization assays. The antibodies with somatically mutated V_H_ regions bound to stabilized CON-S chimeric DS.SOSIP.664. This binding was expected since CON-S gp140 was the immunogen that elicited DH501 in macaques [37]. Additionally, the antibodies with somatically mutated V_H_ regions bound to a heterologous Env gp140, BG505 6R.SOSIP.664 (**Fig 1C**). In contrast, antibodies lacking V_H_ somatic mutations alone or lacking V_H_ and V_L_ somatic mutations did not bind to either trimeric HIV-1 Env gp140 (**Fig 1C**). The binding of each antibody to native fusion-competent Env gp160 on JR-FL pseudoviruses was examined in neutralization assays. JR-FL was treated with kifunensine to restrict glycosylation to Man_9_GlcNAc_2_ [42, 43]. Man_9_GlcNAc_2_ enrichment of JR-FL makes it sensitive to DH501 neutralization, while not changing its overall neutralization sensitivity [37]. DH501 potently neutralized JR-FL glycosylated with Man_9_GlcNAc_2_ (IC50 = 0.09 μg/mL), whereas the UCA did not (**Fig 1D**). The DH501 antibody with somatic mutations only in the VH also potently neutralized the virus (IC50 = 0.06 μg/mL) (**Fig 1D**). In concordance with Env gp140 binding, antibodies with somatic mutations only in the V_L_ failed to neutralize HIV-1 (**Fig 1D**). Thus somatic mutation of the DH501 heavy chain was necessary and sufficient for conferring Env binding and HIV-1 neutralization.

### Crystal structure of the DH501 UCA

**Fig 2:**
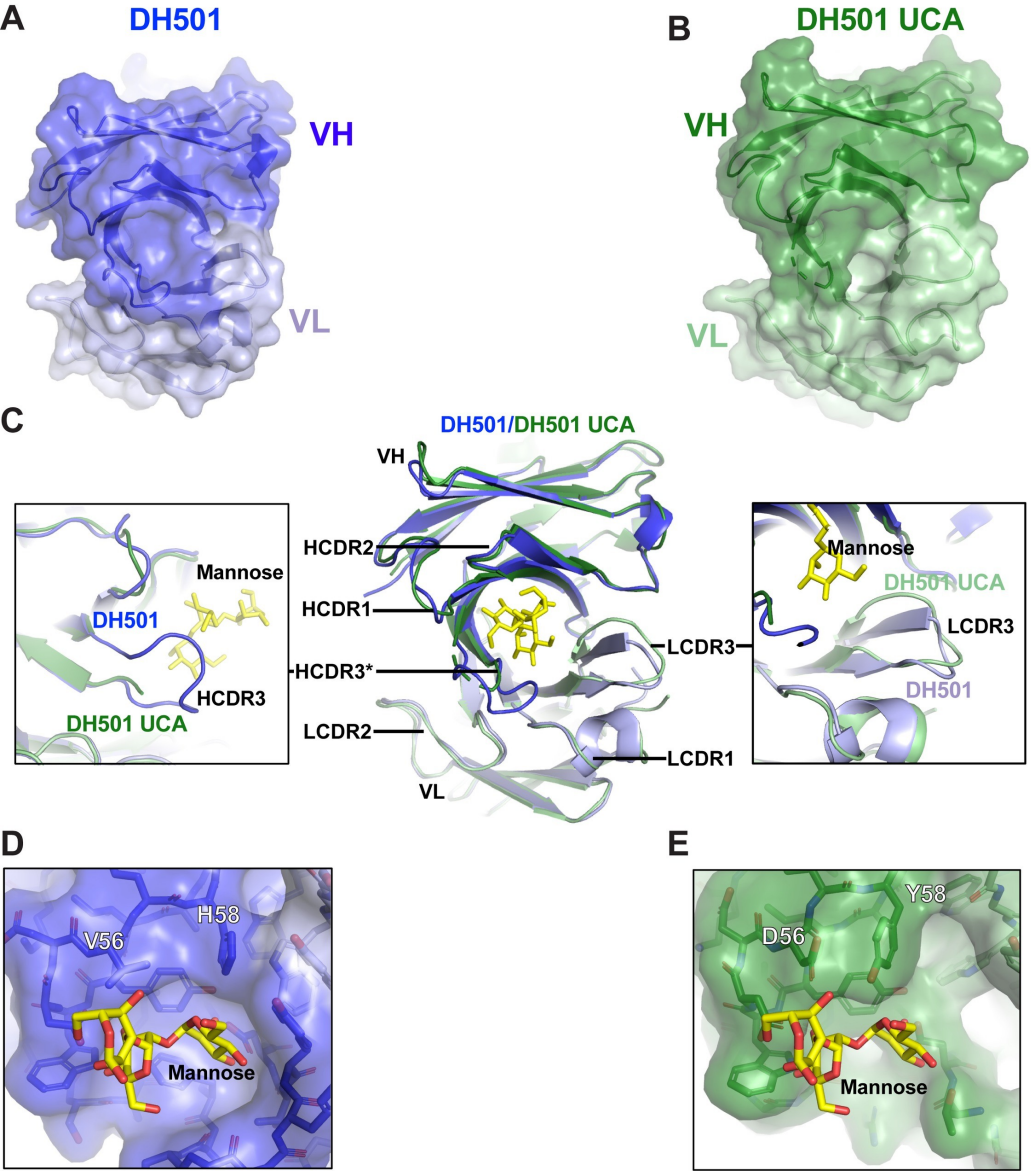
The crystal structure of the DH501 UCA. (**A,B**) Top view of the crystal structures of (**A**) DH501 (blue; PDB: 5T4Z) and the inferred (**B**) DH501 UCA (green; PDB: 6P3B). The variable region of the heavy chain (V_H_) is shown in dark colors and the variable region of the light chain (V_L_) is shown in the light color. Portions of the HCDR3 in DH501 UCA were disordered, therefore, HCDR3 was omitted in the surface representation of the Fv structure. **(C)** A superposition of the Fv regions of the DH501 UCA (dark green heavy chain, light green light chain) and mature DH501 (dark blue/light blue). Inserted into the DH501 glycan-binding cavity is the stick structure of the terminal mannose on the D2 arm of Man_9_GlcNAc_2_ (yellow sticks). Zoomed in views of the HCDR3 and LCDR3 are shown in the boxes on the left and right side respectively. The disordered HCDR3 in DH501 is marked with asterisks. **(D, E)** Zoomed-in view of the glycan-binding cavity in (**D**) DH501 and (**E**) DH501 UCA antigen binding sites. Somatic mutation at position 56 and 58 are labeled with the corresponding amino acids. Note the side chain of Y58_HFWR3_ pointing down into the glycan-binding cavity as compared to H58 that forms a smooth, round-shaped roof of the cavity with its side chain oriented differently. Image created in Pymol [41].

We hypothesized that the V_H_ somatic mutations could orient the CDRs such that the glycan-binding cavity could be formed. To determine whether somatic mutation was required for the formation of the glycan-binding cavity, we determined the crystal structure of the unliganded DH501 UCA Fab to 2.10 Å resolution (**Fig 2A and 2B**). The structure had one Fab in the asymmetric unit (AU) compared to two Fab/AU for the somatically-mutated DH501 Fab in both its unliganded and liganded structures [Fig 2A; 37]. In a superposition, the DH501 UCA Fv regions had a RMSD of 0.70 Å compared to affinity-matured DH501 (**Fig 2C**). The HCDR3 was disordered in the DH501 UCA structure (**Fig 2C**). All six CDRs oriented to a moderately large solvent channel, possibly conferring steric freedom in contrast to both mature DH501 structures where the majority of residues in the paratopes of both Fabs in the AUs were involved in non-crystallographic and/or crystallographic contacts [37]. This difference in crystal formation may explain the difficulty in resolving the DH501 UCA HCDR3.

**Fig 3:**
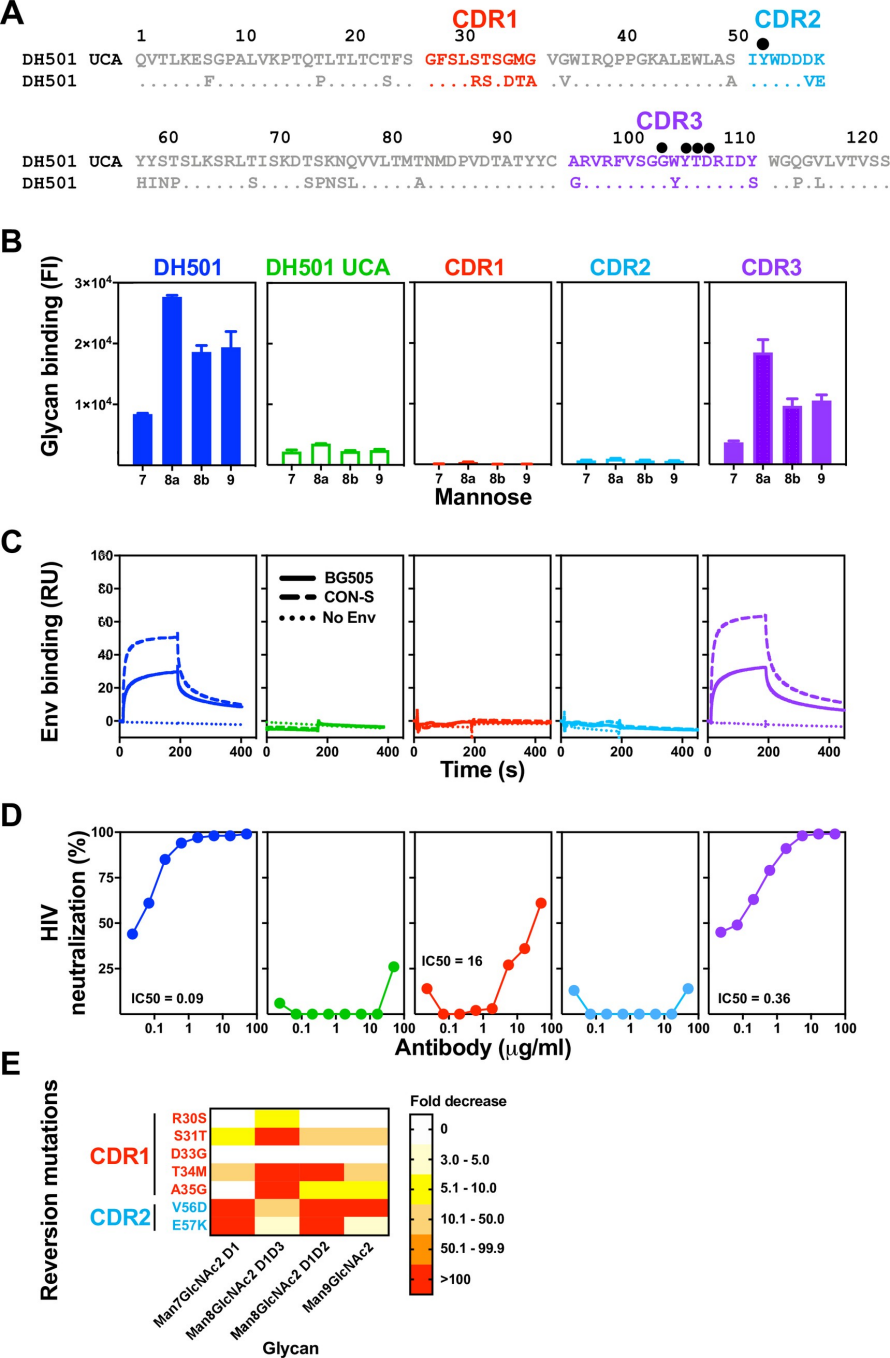
Somatic mutation of HCDR1 and HCDR2 is required for glycan reactivity and HIV-1 Env reactivity. **(A)** ClustalW alignment of amino acids of DH501 and DH501 UCA V_H_. HCDRs are denoted based on the IMGT definition and color-coded. Identical residues are shown as dots. Amino acids resulting from somatic mutation are specified for DH501. Black circles above the sequence alignment denote amino acids that contact glycan in the crystal structure of DH501 in complex with Man_9_GlcNAc_2_. **(B)** Antibody binding to glycan was assessed for Man_7_GlcNAc_2_ D1 (7), Man_8_GlcNAc_2_ D1D3 (8a), Man_8_GlcNAc_2_ D1D2 (8b), Man_9_GlcNAc_2_ (9). Binding was assessed for DH501 with the HCDR1 (red), HCDR2 (light blue), or HCDR3 (purple) somatic mutations reverted to the UCA sequence. Column titles indicate the antibody analyzed in each functional assay. Antibodies are color-coded the same in **B**-**D**. The mean and standard error are shown for independent triplicate experiments. Positive binding based on negative control antibody binding is shown as a filled bar. Open bars indicate negative binding values. Positivity thresholds for 7, 8a, 8b, and 9 are 0.18x10^4^, 0.32x10^4^, 0.19x10^4^, 0.18x10^4^ respectively. **(C)** Trimeric HIV-1 envelope reactivity of DH501, DH501 UCA, and HCDR-reverted antibodies. IgG binding to CON-S SOSIP (dashed line) or BG505 6R.SOSIP.664 (solid line) was determined by SPR. IgG binding response measured with the buffer only control is represented as dotted lines. Representative data from two independent experiments are shown. **(D)** *In vitro* HIV-1 neutralization of kifunensine-treated, Man_9_GlcNAc_2_-enriched JR-FL in the TZM-bl assay. For antibodies with detectable neutralization titers, the IC50s in μg/mL are shown. **(E)** Single somatic mutations in the HCDR1 and 2 were reverted to the UCA sequence and binding to the free glycans as in **B** was assessed. The reversion mutations are color-coded based on their location shown in **A**. The heatmap shows the fold decrease in binding upon reversion of the single mutation. The ranges of fold decrease in glycan binding are color-coded as indicated in the legend.

In the portions of the UCA that were ordered, we analyzed how somatic mutation affected the structure of the glycan-binding cavity. In examining the mutations accrued with affinity maturation in the DH501 lineage, a cluster of six mutations was found to have occurred in the C” beta strand across the junction of the IMGT-defined HCDR2 and FWR3 (**Fig 3A**). The side chains of V56_HCDR2_ and H58_HFWR3_ in the V_H_ somatic mutation cluster were oriented toward bound mannose moieties [37]. Though they were not involved in specific polar interactions with glycan, they were critical in forming the glycan-binding pocket with good shape complementarity to the ligand. The analogous residues in the DH501 UCA, D56_HCDR2_ and Y58_HFWR3_ (**Fig 2E**), were appropriately positioned to create the rudimentary glycan-binding pocket. However, the wall of the cavity formed by UCA HCDR2 was collapsed inward as compared to the mature DH501 (**Fig 2D and 2E**). In total, the initial V_H_DJ_H_ recombination event formed an initial cavity whose shape near the HCDR2 and HFWR3 was optimized by somatic mutation.

### DH501 HCDR1 and HCDR2 somatic mutations are required for glycan reactivity and Env trimer recognition

The crystal structure of the DH501 UCA provided insight for the role of HCDR1 and HCDR2 in the formation of the glycan binding cavity. However, the disordered HCDR3 limited our understanding of the role of somatic mutations in glycan reactivity in this HCDR. In an effort to define the minimal mutations that would be necessary for a vaccine to elicit glycan-reactive antibodies, we refined the somatic mutation reversion experiments by examining individual CDRs and individual FWRs. We focused only on the heavy chain since DH501 H paired with UCA L was still able to bind to glycans and neutralize HIV-1 (**Fig 1**). This result indicated that the most important somatic mutations were in the heavy chain. The heavy chain of DH501 possessed 28 (23%) amino acid changes compared to its putative precursor immunoglobulin chain (**Fig 3A**). Ten of the amino acid changes were within the IMGT-defined CDR loops and the remaining 18 changes were present in FWRs. We reverted the five somatic mutations in HCDR1 (**Fig 3A**) and measured glycan reactivity, trimeric SOSIP gp140 binding, and HIV-1 neutralization. Although none of the somatic mutations in HCDR1 directly contact glycan in the crystal structure, reversion of the HCDR1 mutations abrogated mannose reactivity (**Fig 3B**). HCDR2 contains one glycan contact residue [37], which is germline encoded. Despite the glycan contact residue being present, HCDR2 reversion of the two somatic mutations in HCDR2, V56 and E57, (**Fig 3A**) also eliminated glycan reactivity. The HCDR3 results from V_H_DJ_H_ recombination, and was inferred here with the Cloanalyst software using a Bayesian probability statistical model [44]. As with any inference there is a degree of uncertainty in the V-D and D-J junctions formed by the recombination event. The crystal structure of DH501 indicated that G100a, Y100c, D100e, all contacted glycan directly, with T100d providing contacts through water molecules [37]. Interestingly, none of these contact residues were somatic mutations based on our UCA inference (**Fig 3A**). Nonetheless, the inferred UCA HCDR3 did differ from DH501 by three amino acids (**Fig 3A**). When all three of these amino acids were reverted in DH501 to the inferred UCA sequence, glycan reactivity was maintained (**Fig 3B**). Each of the CDR-reverted antibodies were also tested for HIV-1 envelope engagement. Similar to glycan reactivity, HCDR1 and HCDR2 somatic mutations were required for antibody binding to recombinant soluble Env trimers, CON-S DS.SOSIP and BG505 6R.SOSIP.664, and for neutralization of kifunensine-treated JR-FL pseudovirus (**Fig 3C and 3D**). In contrast, somatic mutation of HCDR3 was not required for either of these functions (**Fig 3C and 3D**). Taken together, somatic mutation of HCDR1 and HCDR2 was required for antibody function, but somatic mutation of HCDR3 was not required.

### HCDR2 mutations V56D and E57K are required for Man7, 8, and 9 reactivity

Reversion of all HCDR1 or all HCDR2 somatic mutations dramatically reduced glycan reactivity. We generated single reversion mutations for each of the somatic mutations in HCDR1 and HCDR2 to determine which somatic mutations were functionally important. The binding to glycan by each single mutant was measured and compared to the wildtype DH501 antibody to generate relative binding values. Interestingly, not all somatic mutations within the HCDR1 were required for glycan binding. Across the five mutations within HCDR1, only S31T and T34M conferred decreased binding to all four glycans tested (**Fig 3E**). Man_8_GlcNAc_2_ recognition was hindered the most by reverting the S31 and T34 to germline. A35 in the HCDR1 also contributed to Man_8_GlcNAc_2_ D1D3 binding, but had minimal effect on binding to other glycans (**Fig 3E**). The two mutations V56 and E57 were shown in the crystal structure to form the glycan-binding cavity [Fig 2; 37]. Reversion of both of these single mutations conferred dramatic reductions in glycan binding. The reversion mutation V56D reduced Man_7_GlcNAc_2_, Man_8_GlcNAc_2_D1D2, and Man_9_GlcNAc_2_ binding by greater than 100-fold (**Fig 3E**). E57K reduced binding by greater than 100-fold for Man_7_GlcNAc_2_ and Man_8_GlcNAc_2_ D1D2 (**Fig 3E**). Interestingly, reversion of the HCDR2 somatic mutations had a moderate effect on Man_8_GlcNAc_2_ D1D3 binding. Instead, DH501 binding to this glycan appeared to be mostly determined by the S31T, T34M, and A35G mutations in HCDR1. These results indicate HCDR2 somatic mutations determined binding to Man_7_GlcNAc_2_ D1, Man_8_GlcNAc_2_ D1D2, and Man_9_GlcNAc_2_, whereas HCDR1 mutations mostly contributed to Man_8_GlcNAc_2_ D1D3 binding.

### The essential role of putative germline-encoded amino acids in DH501 glycan binding

**Fig 4:**
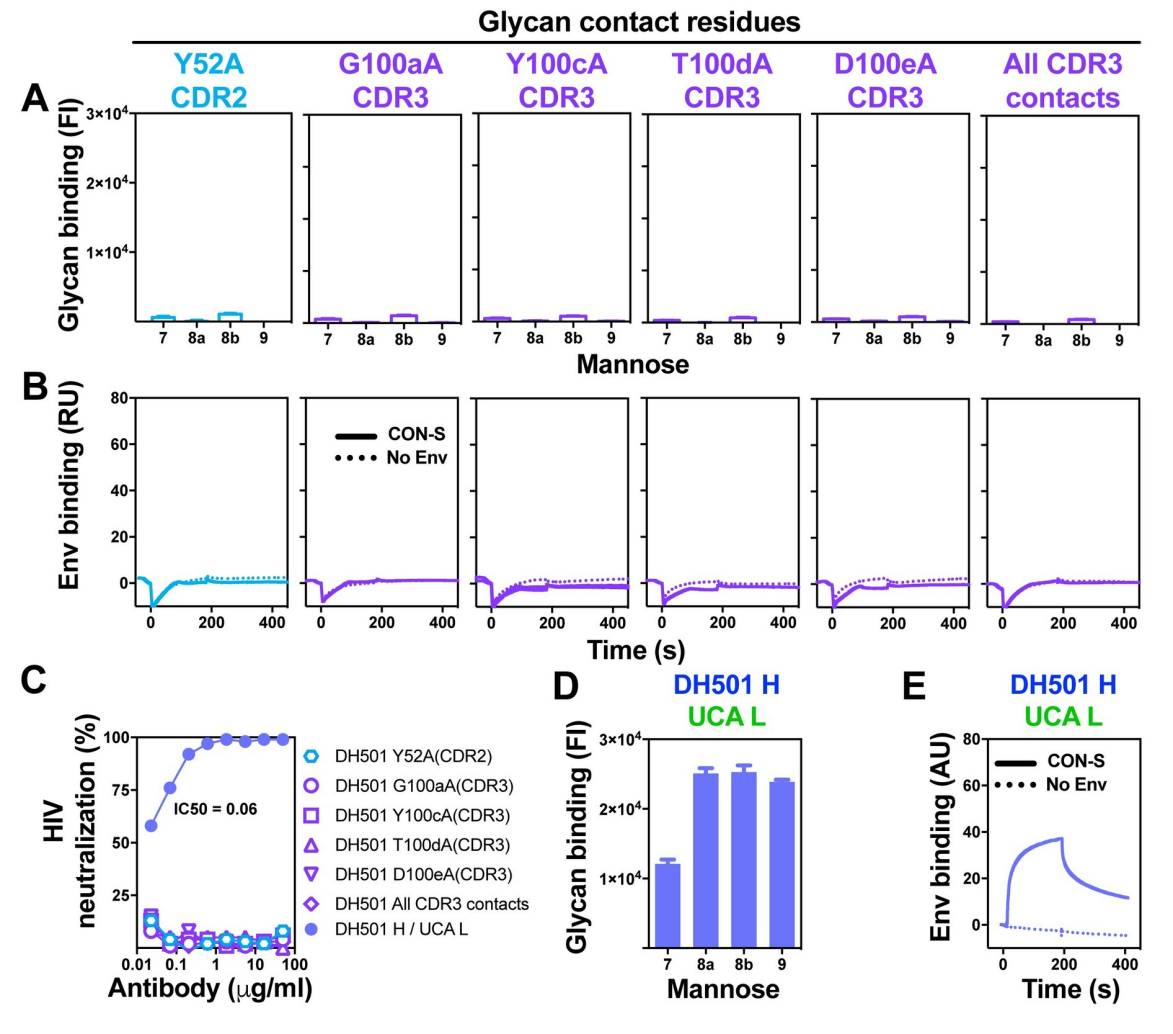
Germline-encoded residues in the HCDR2 and HCDR3 mediate glycan contacts and are essential for free glycan binding. Alanines were substituted for germline-encoded amino acids identified in the DH501 structure to contact glycan (circles in **Fig 3A**; [37]). HCDR2 and HCDR3 substitutions are shown in cyan and purple respectively, with amino acid position indicated above each column. (**A**) Glycan binding, (**B**) HIV-1 CON-S SOSIP gp140 binding, and (**C**) Man_9_GlcNAc_2_-enriched JR-FL neutralization activity for each antibody is shown as in **Fig 3A–3C**. The somatically mutated antibodies are color-coded the same in **A-C**. **(C-E) (C)** Man_9_GlcNAc_2_-enriched JR-FL neutralization activity, (**D**) Glycan binding, and (**E**) HIV-1 CON-S SOSIP gp140 binding by positive control antibody DH501 H/ UCA L. Positive glycan binding based on negative control antibody binding is shown as a filled bar. Open bars indicate negative binding values. Positivity threshold for 7, 8a, 8b, and 9 was 0.18x10^4^ for each glycan.

The crystal structure of DH501 in complex with mannose glycan showed five amino acids that contacted the terminal mannose residues on the D2 arm of the glycan. Four of the contact residues were in the HCDR3 and the remaining residue was in the HCDR2. All five of the amino acids were encoded by germline nucleotide sequence based on our inference, which has a degree of uncertainty. To determine whether all of these contacts were required for glycan reactivity, we individually changed each residue to alanine. Antibodies encoding Y52aA, G100aA, Y100cA, T100dA, or D100eA lost glycan binding (**Fig 4A**). Likewise, a mutant DH501 bearing all four HCDR3 alanine changes also lacked glycan binding (**Fig 4A**). Each of the glycan contact residues was also required for antibody binding to recombinant HIV-1 Env gp140 (**Fig 4B**). This lack of Env reactivity translated to a loss of neutralization activity against kifunensine-treated JR-FL virus (**Fig 4C**). Therefore, changing a single germline-encoded amino acid eliminated antibody function. While the germline-encoded amino acids were required for binding glycan, they were not sufficient for monomeric IgG binding to glycan (**Fig 1B**). Avidity did not improve glycan binding by the DH501 UCA either, since when overexpressed in multiple copies on the cell surface the DH501 UCA did not bind to Man_9_GlcNAc_2_ (**S2 Fig**).

### HCDR2 amino acids at position 54, 55, and 57 contribute to envelope recognition

**Fig 5:**
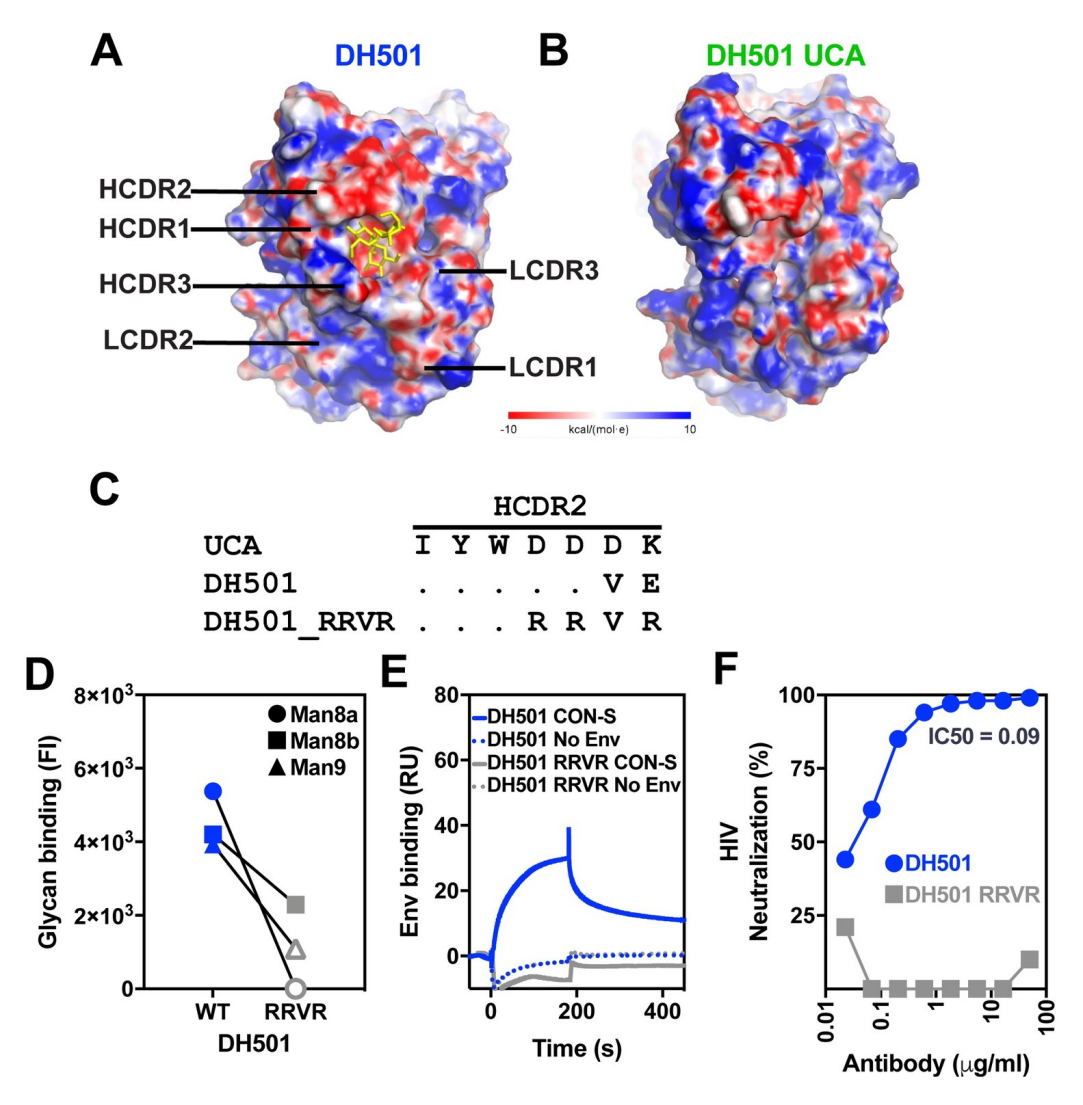
The negatively charged amino acid patch in HCDR2 is required for HIV-1 Env trimer binding. **(A, B)** The electrostatic surface potential of (**A**) DH501 and (**B**) the DH501 UCA. The glycan binding pocket of the DH501 antibody has a negative charge. The terminal mannose residues of the D2 arm of Man_9_GlcNAc_2_ are shown in yellow. The electrostatic potentials are oriented with the paratope facing towards the viewer. (**C**) Amino acid alignment of HCDR2 of DH501 and the DH501 UCA. Dots indicate identical residues. The negatively-charged residues within the HCDR2 of DH501 were changed to positively-charged arginine residues (RRVR). (**D**) DH501 wildtype (WT) and the DH501_RRVR variant (RRVR) IgG binding to high mannose glycans in a custom luminex assay. The mean of 3 independent experiments is shown. Positive glycan binding values are shown as filled symbols. Positivity threshold for 7, 8a, 8b, and 9 was 1.8x10^3^ for each glycan. (**E**) DH501 and DH501_RRVR IgG binding to soluble, trimeric CON-S SOSIP gp140s (solid line). Dashed lines show binding magnitudes in the absence of Env. Images are representative of two independent experiments. **(F)** DH501 and DH501_RRVR IgG *in vitro* HIV-1 neutralization of Man_9_GlcNAc_2_-enriched JR-FL.

Analysis of the electrostatic surface potentials of DH501 and DH501 UCA showed both antibodies possessed a negatively-charged HCDR2 (**Fig 5A** and **5B**). For the DH501, a cluster of negatively-charged amino acids D54, D55, and E57 contributed to the negative charge of the HCDR2 (**Figs 3A**, **5A** and **5C**). The negative charge was also present in the DH501 UCA HCDR2 due to the presence of amino acids D54, D55, and D56 (**Figs 3A**, **5B** and **5C**). To determine whether these amino acids in the HCDR2 were important for DH501 function, we introduced D54R, D55R, and E57R substitutions in the HCDR2 (**Fig 5C**). DH501 containing these mutations (termed DH501_RRVR) bound weaker to Man_8_GlcNAc_2_ and Man_9_GlcNAc_2_ glycans than wildtype DH501 (**Fig 5D**). This result was consistent with the knockout of glycan binding found with the single E57K change (**Fig 3E**). DH501_RRVR lost reactivity with recombinant trimeric HIV-1 Env gp140 and native trimeric Env on virions (**Fig 5E** and **5F**). Hence, the D54, D55, and E57 was required for optimal binding to Env, Man_8_GlcNAc_2_, and Man_9_GlcNAc_2_.

### Somatic mutations within the framework regions confer antibody function

**Fig 6:**
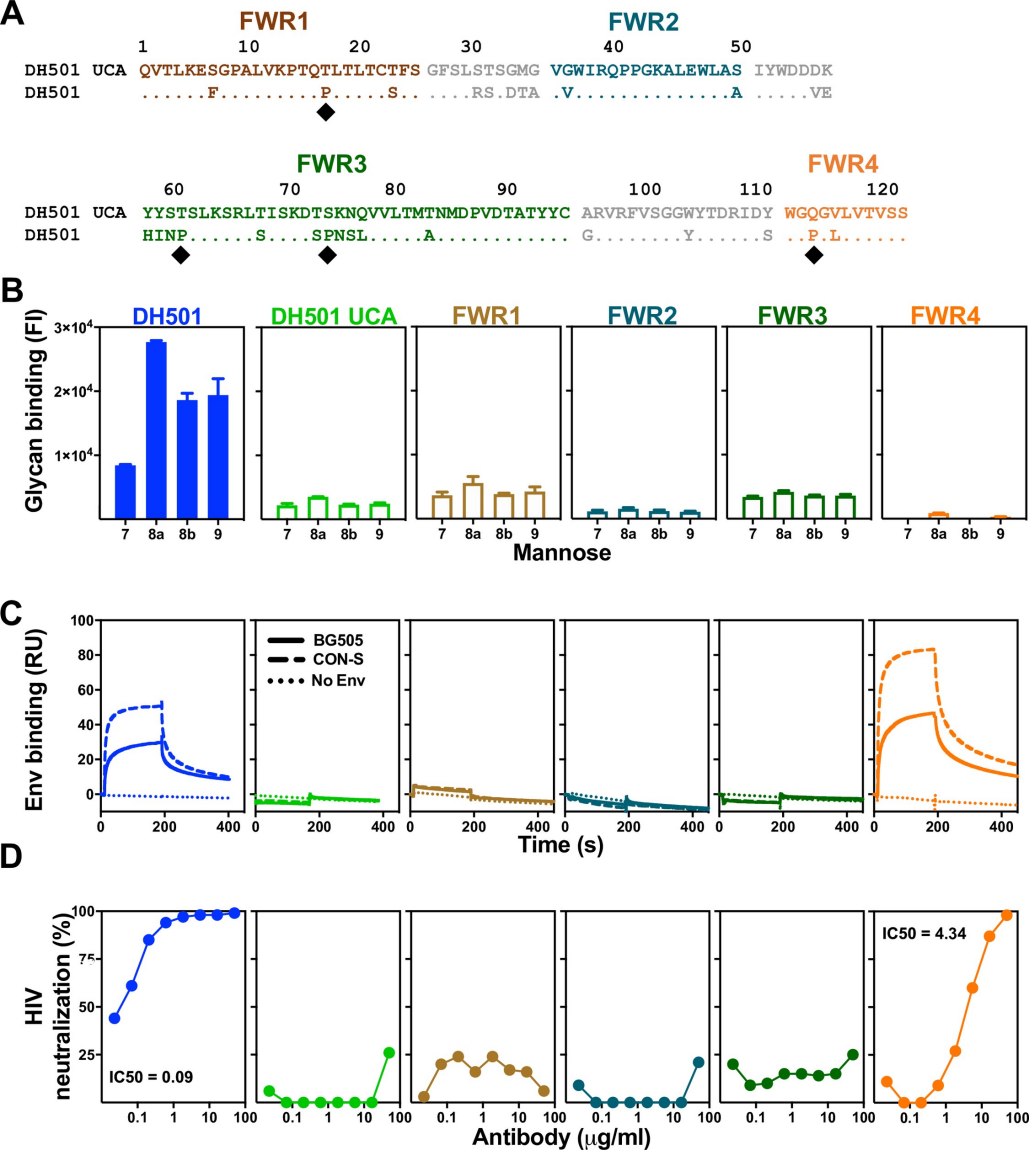
Somatic mutation of HFWR1, HFWR 2, and HFWR 3 is required for glycan reactivity and HIV-1 Env reactivity. (**A**) ClustalW alignment of amino acids of DH501 and DH501 UCA VH. Identical residues are shown as dots. HFWRs are denoted based on the IMGT definition and color-coded. Black diamonds below the sequence alignment denote somatic mutations that introduce a proline substitution in HFWRs. **(B)** Binding of DH501 HFWR reverted antibodies to Man_7_GlcNAc_2_ D1 (7), Man_8_GlcNAc_2_ D1D3 (8a), Man_8_GlcNAc_2_ D1D2 (8b), Man_9_GlcNAc_2_ (9). The reverted antibodies are color-coded the same throughout **B**-**D**. Mean values + standard error are shown for triplicate experiments. Positive glycan binding based on negative control antibody binding is shown as a filled bar. Open bars indicate negative binding values. Positivity thresholds for 7, 8a, 8b, and 9 are 0.6x10^4^, 0.90x10^4^, 0.67x10^4^, 0.64x10^4^ respectively. **(C)** DH501, DH501 UCA, and HFWR-reverted IgG binding to CON-S SOSIP gp140 Env trimers (dashed line), BG505 6R.SOSIP.664 Env trimers (solid line), or buffer only was determined by SPR. Representative data from two independent experiments are shown. **(D)** *In vitro* HIV-1 neutralization of kifunensine-treated, Man_9_GlcNAc_2_-enriched JR-FL in the TZM-bl assay. IC50 neutralization titers are shown in μg/mL as in **Fig 4D**.

For most antibodies, antigen engagement is primarily governed by the CDRs of the antibody [45]. However, for HIV-1 broadly neutralizing antibodies, somatic mutations in the FWRs are critical for antibody neutralization activity [46–49]. We determined the effects of HFWR somatic mutations in DH501 on glycan reactivity by reverting the somatic mutations in FWR1, 2, 3, and 4 individually. HFWR1 possessed three somatic mutations including the introduction of a phenylalanine and proline (**Fig 6A**). HFWR2 contained two somatic mutations. HFWR3 possessed eleven mutations, which was the most mutations among all of the HFWRs. Lastly, HFWR4 included two somatic mutations (**Fig 6A**). Reversion of the somatic mutations in HFWR1, 2, or 3 knocked down glycan reactivity to the levels observed with the DH501 UCA (**Fig 6B**). Similarly, binding to recombinant soluble Env trimers and kifunensine-treated JR-FL neutralization was abrogated by reverting the HFWR1, 2, or 3 somatic mutations (**Fig 6C** and **6D**). Conversely, reversion of HFWR4 did not eliminate all antibody function. Reverting the proline to glutamine and leucine to valine in the HFWR4 eliminated glycan binding (**Fig 6B**), but did not eliminate trimeric recombinant Env binding (**Fig 6C**). HIV-1 neutralization was still detectable, but had decreased from 0.09 μg/mL to 4.34 μg/mL. Thus, DH501 with reverted HFWR4 somatic mutations had reduced function, but its function was not reduced to the same level as the UCA.

**Fig 7:**
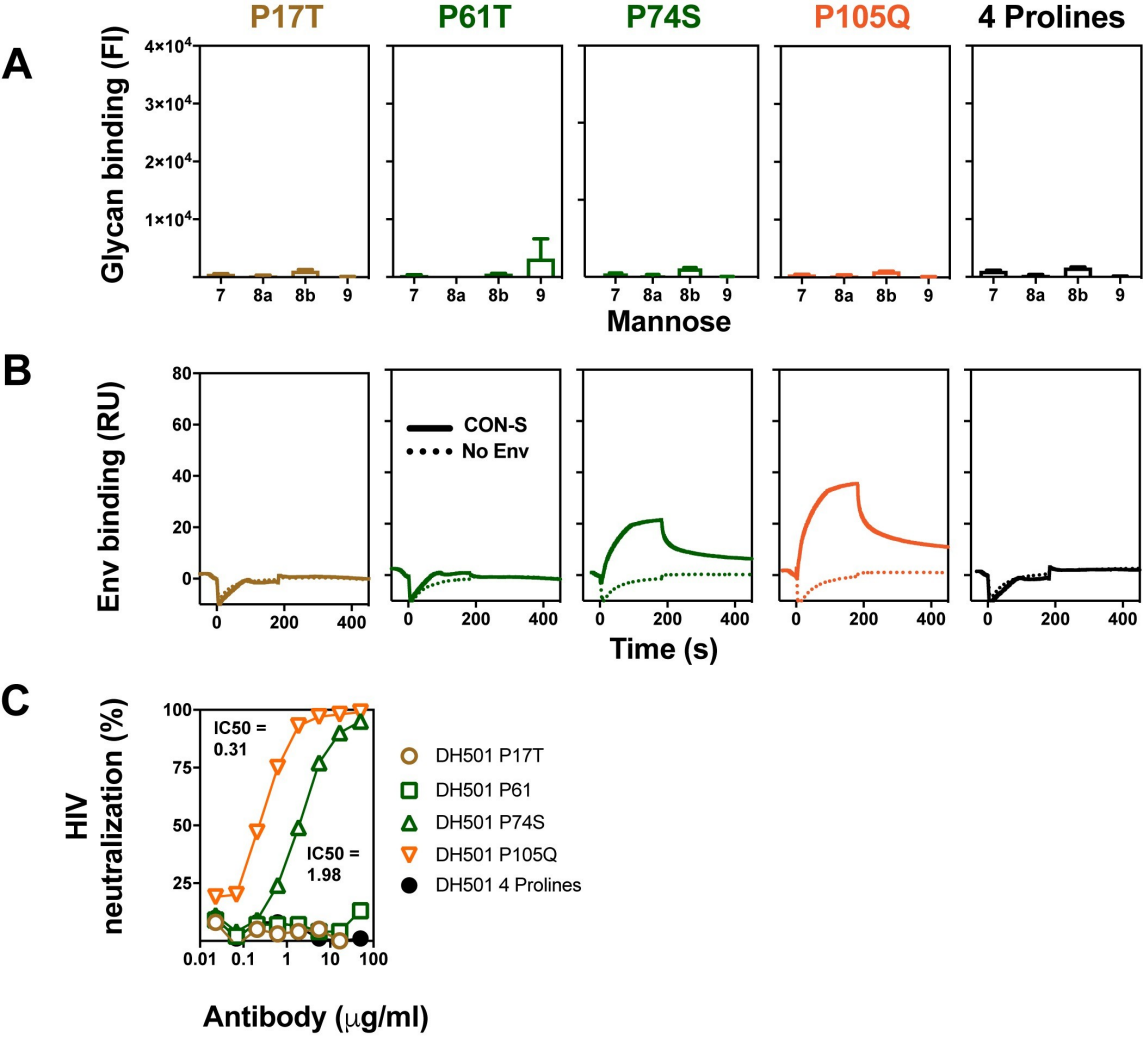
HFWR somatic mutations encode proline amino acids that are critical for antibody function. **(A)** High mannose glycan reactivity for DH501 IgG variants where framework region prolines were reverted to their germline-encoded amino acid. Prolines selected for reversion are indicated with diamonds in **Fig 6A**. Prolines were reverted individually, or all four prolines were reverted in the same antibody (4 prolines). Positive glycan binding based on negative control antibody binding is shown as a filled bar. Open bars indicate negative binding values. Positivity threshold for 7, 8a, 8b, and 9 was 0.18x10^4^ for each glycan. DH501 H/UCA L which includes all four prolines was used as a positive control as shown in **Fig 4**. (**B**) CON-S SOSIP binding by SPR for each of the DH501 proline revertants. Antibody binding to buffer without envelope is shown by a dotted line (No Env). (**C**) DH501 proline-revertant antibody neutralization of Man_9_GlcNAc_2_-enriched HIV-1 JR-FL infection of TZM-bl cells.

Analysis of the DH501 FWR amino acid sequence showed an abundance of somatic mutations that encoded prolines. Since prolines result in kinks in the polypeptide chain, we reasoned that these mutations may be necessary for orienting the CDRs to create a stable glycan-binding cavity [50, 51]. Therefore, we mutated the four prolines individually or in combination to determine their functional significance. DH501 glycan reactivity was lost upon reversion of each proline to UCA sequence (**Fig 7A**). P74 and P105 could be reverted to UCA sequence without completely knocking out Env reactivity and virus neutralization (**Fig 7B** and **7C**). In contrast, P17 in FWR1 and P61 in FWR2 were required for Env reactivity and virus neutralization (**Fig 7B** and **7C**). Thus, all of the prolines were functionally important for glycan binding, with P17 and P61 being the principal determinants of antibody function.

We attempted to define the minimal number of somatic mutations required for glycan binding. We created four variants of DH501 that contained different combinations of framework prolines somatic mutations and somatic mutations from CDR1, CDR2, FWR2, and FWR3. However, none of the four combinations resulted in a DH501 variant that was glycan reactive (**S3 Fig**).

### Somatic mutation of DH501 reduces the thermostability of the antigen binding fragment

**Fig 8:**
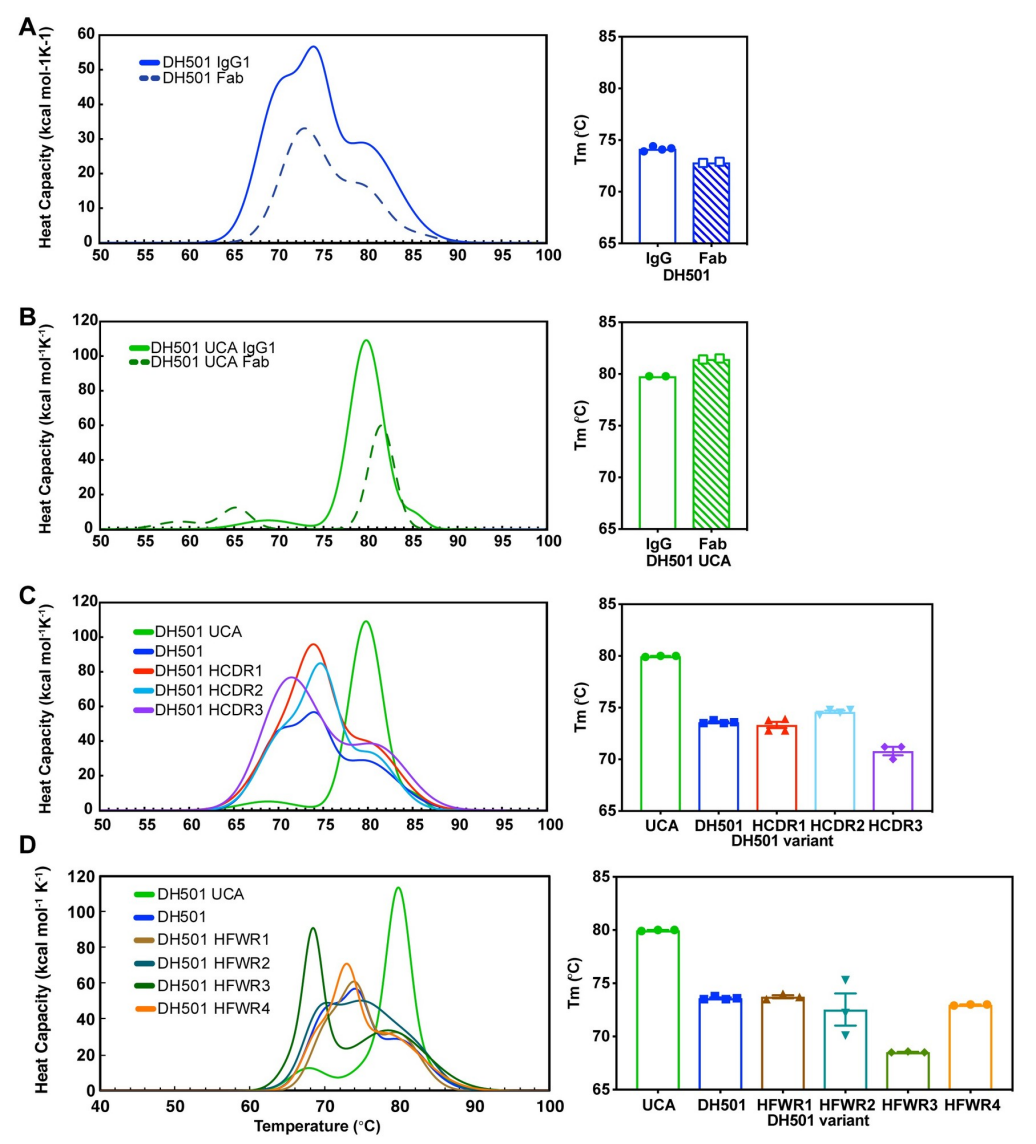
Somatic mutation reduces the thermostability of DH501. **(A, B)** The melting temperature (Tm) of (**A**) DH501 and the (**B**) DH501 UCA. The Tm of IgG (solid line) and Fab (dashed line) was determined by differential scanning calorimetry. The melting curves (left) show the melting transitions and the Tm for IgG and Fab is shown in the bar graph (right). The bar graph symbols represent the independent measurements with the bar showing the mean of the replicates. **(C,D)** The melting curves and Tm of (**C**) HCDR-reverted and (**D**) HFWR-reverted DH501 IgG antibodies. The bar graph symbols represent independent measurements with the bar showing the mean of the replicates.

Since multiple somatic mutations were critical for antibody function, we hypothesized that they must exert a global effect on the paratope of DH501. To determine whether somatic mutation affected the overall folding and stability of DH501, we compared the melting temperatures (T_m_) of the DH501 UCA and DH501 antibodies using differential scanning calorimetry. The full-length IgG of the DH501 UCA had a T_m_ of 79.8°C, which was 5.7°C higher than the T_m_ of the somatically-mutated DH501 IgG (**Fig 8A** and **8B**). In contrast to the DH501 UCA, the melting curves of the DH501 IgG possessed multiple transitions indicating multiple domains in the antibody melted at different temperatures. To focus on the differences in T_m_ that were due to the Fab of DH501 and its UCA we produced recombinant Fabs and measured their melting temperatures. The DH501 UCA Fab had a T_m_ that was 8.6°C higher than the somatically mutated DH501 Fab—confirming the results observed with IgG (**Fig 8A** and **8B**). While IgG tends to have transitions associated with Fab, CH2, and CH3 domains [52], DH501 was unique in that the Fab fragment of the antibody melted with multiple transitions as well. Since the CH1 and light constant regions were the same between the DH501 and DH501 UCA antibodies, the multiple transitions are due to differences in melting of the variable region of the Fab domain [53]. We examined whether the somatic mutations in the HCDRs or HFWRs led to the increase in instability of the DH501 IgG. We reverted the somatic mutations in each HCDR and each HFWR individually and measured their T_m_. Despite reversion of each HCDR or HFWR, the T_m_ did not increase to the temperature of the DH501 UCA (**Fig 8C and 8D**). Thus, no one set of somatic mutations in the FWRs or CDRs mediated the reduction in stability; instead the collective set of somatic mutations led to the conformational flexibility of the variable region (**Fig 8C and 8D**).

### Neutralization by glycan-dependent broadly neutralizing HIV-1 antibodies requires light chain mutation events

**Fig 9:**
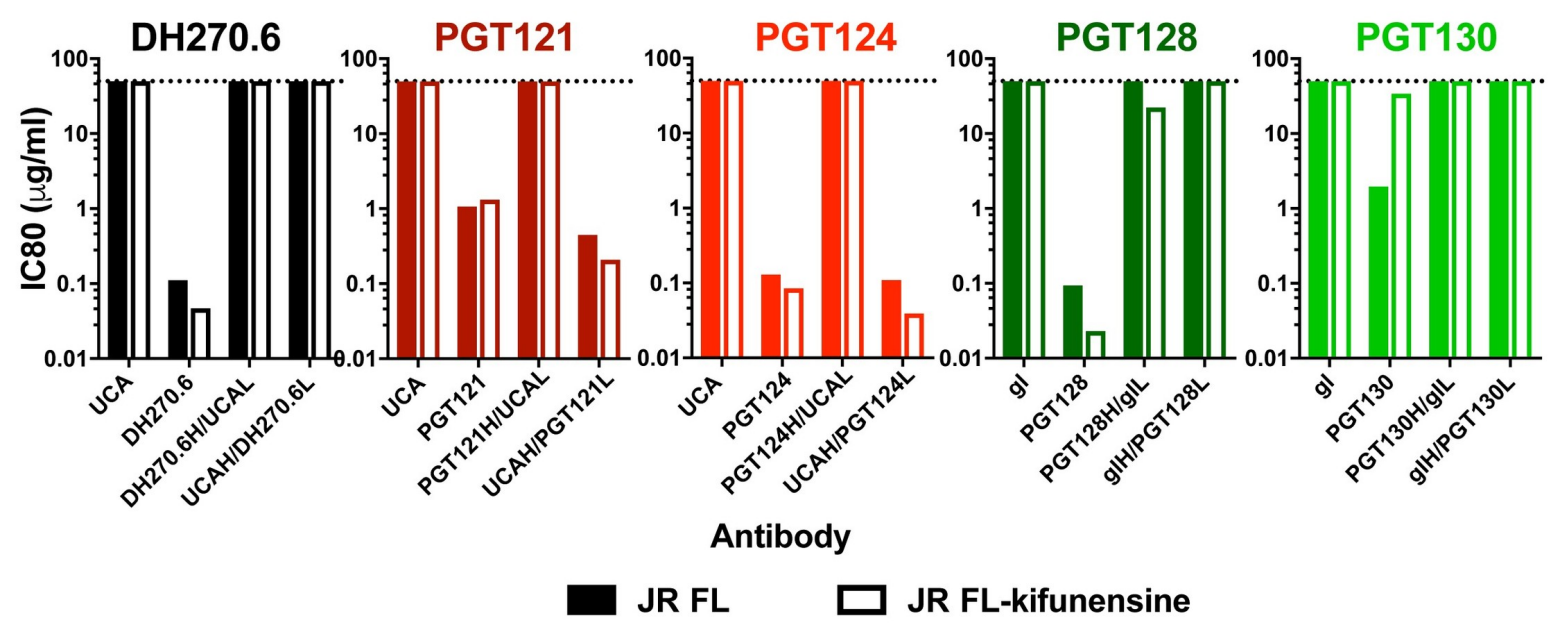
Light chain somatic mutation is required for glycan reactivity of V3-glycan HIV-1 broadly neutralizing antibodies. *In vitro* HIV-1 pseudovirus neutralization of JR-FL (filled bars) and kifunensine-treated (Man_9_GlcNAc_2_-enriched; open bars) JR-FL in the TZM-bl neutralization assay. Neutralization titers are shown in μg/mL as the concentration that inhibits 80% of virus replication (IC80). The antibody tested for neutralization is listed in the graph title. Variants of each antibody are listed on the x-axis. The unmutated common ancestors (UCA) of DH270 and PGT121 lack somatic mutation. For PGT128 the previously published germline reverted antibody (gl) was characterized [57]. PGT121 and PGT124 (reds) are from the same lineage and have the same UCA shown. PGT128 and PGT130 (greens) are clonally related and have the same germline (gl) antibody shown [20, 57].

DH501 showed a strong dependence on heavy chain somatic mutations for recognition of the HIV-1 envelope V3-glycan site. We determined whether V3-glycan-specific broadly neutralizing HIV-1 antibodies also required only heavy chain somatic mutation. Understanding the role of somatic mutation in each immunoglobulin chain of glycan-dependent broadly neutralizing antibodies would help focus vaccine design efforts on a particular immunoglobulin chain. PGT121, PGT128, and DH270 have characteristic long HCDR loops, which crystal structures have shown contact glycan and envelope peptide [27, 54–56]. To delineate the role of somatic mutation in neutralization activity, we expressed five V3-glycan antibodies with either the V_L_ or V_H_ reverted back to germline sequence. Two of the antibodies were from the PGT128 lineage (PGT128 and PGT130) [20], two of the antibodies were from the PGT121 lineage (PGT121, and PGT124) [20], and the last antibody was from the DH270 lineage [22]. Neutralization activity was assessed against difficult to neutralize HIV-1 isolate JR-FL. Reversion of either the V_H_ or the V_L_ mutations of DH270 abolished JR-FL neutralization when measured as the concentration that inhibits 80% of virus replication (IC80; **Fig 9**). The same requirement for mutation of the V_H_ and the V_L_ was observed for the clonally-related antibodies PGT128 and PGT130 (**Fig 9**). In contrast, V_H_ somatic mutation of PGT121 and PGT124 was dispensable for JR-FL neutralization, and loss of neutralization activity occurred only when the light chain was reverted to germline sequence (**Fig 9**). Kifunensine-treatment to enrich Man_9_GlcNAc_2_ glycosylation on JR-FL resulted in more potent neutralization by mannose-reactive antibodies PGT128, DH270 and PGT124 (**Fig 9 open bars**). The enrichment of Man_8_GlcNAc_2_ or Man_9_GlcNAc_2_ by kifunensine treatment had the opposite effect on PGT121 and PGT130 (**Fig 9 open bars**). The effects of kifunensine treatment on neutralization is consistent with the reported crystal structures and glycan array analyses of these antibodies. PGT128, DH270, and PGT124 all have been shown to bind directly to Man_8_GlcNAc_2_ or Man_9_GlcNAc_2_ [20, 22, 27, 56], whereas PGT121 and PGT130 have been shown to bind processed glycans in addition to high mannose [57, 58]. When we examined neutralization potency by the germline reverted antibodies, enriching for Man_9_GlcNAc_2_ did not change the requirements for somatic mutations for most of the antibodies. The one exception was that kifunensine treatment enabled weak neutralization (IC80 = 22 μg/mL) of JR-FL by PGT128 lacking V_L_ somatic mutations, which was not seen for untreated virus (**Fig 9**). In total, the requirement for V_H_ or V_L_ mutation varied across different glycan-reactive HIV-1 antibody lineages, but was consistent within a given lineage. Additionally, DH501 differed from the broad neutralizing glycan-reactive antibodies in that none of them were solely dependent on heavy chain mutation for neutralization activity despite their use of HCDR loops to mediate glycan and peptide contact.

## Discussion

The investigation of antibody recognition of glycan can elucidate the genetic changes and biochemical features of antibodies that have the potential to clear or prevent infection by human pathogenic viruses. Such human viruses include hepatitis C, HIV-1, and influenza, which all express glycosylated envelope proteins [2, 59]. Here we show that somatic mutations in the V_H_ cooperate with germline-encoded residues to enable glycan reactivity and antigen recognition for the vaccine-induced, macaque HIV-1 antibody DH501. The V_H_ somatic mutations did not make direct antigen contacts, but instead increased the flexibility of the antibody variable region. Moreover, the germline-encoded amino acids that contacted glycan were not somatically mutated showing antibody evolution preserved this solution for glycan contact.

In contrast to DH501, somatic mutation of the heavy chain of V3-glycan HIV-1 broadly neutralizing antibodies (bnAbs) DH270.6, PGT121, PGT124, PGT128, and PGT130 was not always sufficient for antibody function. Rather, the immunoglobulin chain that possessed the rare nucleotide insertion/deletion (indel) event was required [20]. For bnAbs PGT130 and DH270, which lacked such indels, somatic mutations in both heavy and light chains were required [20, 22, 57]. DH501 is the only one of these antibodies that binds to glycan with a deep cavity in its heavy chain paratope [37], perhaps explaining why it was different from some of the human V3-glycan antibodies tested here. These results were significant for vaccine design in that they showed whether somatic mutations in the heavy or light chains were necessary for neutralization activity. Given this information immunogens can be designed that optimally bind and select for amino acid changes in the immunoglobulin chain that was shown to be important.

In the structure of DH501 bound to the terminal mannose residue of Man_9_GlcNAc_2_, the residues that contacted glycan were all germline-encoded [37]. Here, we show that each of the five germline-encoded residues are essential for DH501 reactivity with glycan. The requirement of germline-encoded residues is similar to previous observations for other virus-specific antibodies. For example, the nucleotide sequences of HIV-1 CD4 binding site antibodies encode germline residues Arg71, Trp50, and Asn58, which all make critical contacts within the CD4 binding site of HIV-1 Env [60]. Additionally, multiple neutralizing influenza antibodies are derived from the IGVH1-69 gene segment [61, 62], which facilitates binding to influenza hemagglutinin via germline-encoded residues Ile53 and Phe54 in HCDR2 [63]. The presence of functional germline-encoded amino acids has been hypothesized to be the underlying biological reason for shared gene usage among neutralizing antibodies against influenza and HIV-1 [60, 63]. Prior to any somatic mutation, the DH501 precursor was composed of germline gene segments capable of contacting glycan, suggesting that the usage of V_H_2 and J_H_4 gene segments may be a genetic mechanism to engender an antibody with glycan reactivity. More DH501-like antibodies need to be isolated in order to determine whether V_H_2 and J_H_4 gene segments are a common solution for glycan reactivity. Among the 14 known V3-glycan bnAb lineages that have been isolated from infected humans there has not been a certain V or J gene segment known to have intrinsic glycan affinity. However, there is an abundance of V3-glycan antibody light chains derived from V_K_3-20 or V_K_3-15 recombined with J_K_1 or J_K_3 among the 14 most well-characterized V3-glycan bnAbs. Future studies could explore the biochemical basis for why 6 of 14 V3-glycan bnAb lineages utilize these V_K_ and J_K_ gene segments.

The initial V_H_DJ_H_ recombination event facilitated DH501 glycan reactivity because four of the required germline-encoded glycan contact residues utilized by DH501 were present in its HCDR3. HCDR3 recombination requires joining of V_H_, D, and J_H_ gene segments, which when combined with junctional diversity provides a degree of uniqueness to each heavy chain variable region made by the immune system [64–68]. Next generation sequencing of antibody repertoires has shown 1–6% of heavy chain clonotypes can be shared between two unrelated humans [69]. Therefore, identical or very similar V_H_DJ_H_ recombination events can occur in different humans [70, 71], which results in HCDR3s with the similar functionality [70]. These results suggest that antibodies with HCDR3 sequences similar to DH501 could be observed in multiple unrelated individuals. Future sequencing experiments could investigate the frequency of HCDR3s similar to DH501 among the human population.

The thermostability of the antibody is generally determined by the stability of the Ig fold. Upon somatic mutation, DH501 showed a decrease in thermostability suggesting somatic mutation likely destabilized the Ig fold. This hypothesis is consistent with accumulation of somatic mutations in the FWRs of DH501 V_H_, as the FWRs provide the overall structure of the antibody variable region [72]. Among the somatic mutations within the FWRs, four amino acid changes were prolines. One of these prolines was located at position 61 in the C” β strand [72]. The C” β strand joins the two β sheets of the v-type Ig fold, and would be predicted to be important for the structure of the v-region [72, 73]. Also, the C” strand is outside of the core Ig fold and is known to be more flexible than other beta strands in the Ig fold suggesting its position could be changed upon somatic mutation [72, 73]. A Pro61 somatic mutation also occurs in the HIV-1 CD4 binding site bnAb 3BNC60 [48]. The crystal structure of 3BNC60 showed that Pro61 did not contact Env, but instead caused a kink in the β strand that eliminated hydrogen bonds between C’ and C” β strands [48]. The elimination of the hydrogen bonds reoriented the CDR loop such that it would be predicted to contact HIV-1 envelope [48]. It is conceivable that the four prolines observed in DH501 FWRs, including Pro61, function similarly in DH501. Prolines cause kinks in alpha-helices [51], and are usually not found in β sheets since they lack the appropriate hydrogen bonding pattern [50]. Prolines are also favorable for β turns in which the peptide backbone abruptly changes direction [74]. Together, the presence of four prolines in the β sheets of FWRs suggests they are present due to selection during affinity maturation of DH501, and likely contribute to the structure of the paratope. Indeed, the four prolines were required for glycan reactivity, supporting the notion that they were selected for during affinity maturation. Hence, the role of the prolines in the FWRs of DH501 may be to destabilize the Ig fold such that the FWR β strands can reorient the CDR loops into the positions necessary to form the deep glycan-binding cavity.

This loss-of-function study identified that free glycan reactivity was dependent on many of the heavy chain somatic mutations present in DH501. In contrast to the light chain where all somatic mutations were not required, only the somatic mutations in the HCDR3 could be reverted to putative germline sequence and retain glycan binding. Thus, we found that the addition of the somatic mutations in HCDR1, HCDR2, HFWR1, HFWR2, HFWR3, and HFWR4 to the germline antibody were sufficient for glycan binding. We did not observe a smaller subset of mutations that conferred DH501 glycan binding. This relatively large number of somatic mutations was likely necessary since the somatic mutations changed the overall flexibility of the antigen binding fragment—as opposed to making more productive contacts. It should be noted that binding to free glycan is more sensitive to changes in the antibody sequence than envelope binding. For example, DH501 with the HFWR4 reverted to germline sequence bound to envelope, but lost binding to free glycans. In general, antibody:carbohydrate interactions are thought to be in the micromolar affinity range, compared to nanomolar affinity ranges for antibody:protein interactions [12]. Since antibody:carbohydrate interactions are weak, it is not surprising that the reversion of multiple somatic mutations across the paratope would reduce DH501 glycan binding to levels below the limit of detection.

In summary, this study shows the complex genetic and biochemical events that would need to occur during vaccination for DH501-class anti-glycan antibodies to be routinely elicited. The requirement for a V_H_DJ_H_ rearrangement encoding glycan contact residues, somatic mutations that increase variable region flexibility, and somatic mutations that encode atypical proline residues present challenges for eliciting glycan-dependent antiviral antibodies. We hypothesize vaccine immunogens will need to persist in germinal centers to prolong affinity maturation by somatic mutation in order to overcome these challenges. Nonetheless, these types of glycan-dependent antibodies may be more readily induced than the broadly neutralizing antibodies that require rare nucleotide insertion and deletion events in order to react with glycan-dependent epitopes. Thus, vaccine regimens that use mannosylated immunogens to affinity mature DH501-like antibodies to bind and neutralize diverse HIV-1 isolates warrant further study.

## Materials and methods

### Crystallography

Fab fragments of DH501 UCA were produced recombinantly as previously described [75]. Briefly, Fab chains were generated by PCR using light and heavy chain genes as templates with appropriate primer pairs and cloned into pcDNA3.1/hygro (+) [76]. Recombinant Fabs were produced in Freestyle 293 (Invitrogen) cells by transient transfection then purified using methods described previously [75]. Fabs were further purified via size exclusion chromatography (SEC) using a GE HiLoad 26/60 Superdex 200pg 26/60 column at 3.2 mL/min with a buffer of 10 mM HEPES pH 7.2, 50 mM NaCl, 0.02% NaN_3_. Peak protein-containing fractions were concentrated, buffer exchanged to ddH2O, and brought to 15.0 mg/ml.

All protein samples were tested against commercially available screens (Qiagen, Molecular Dimensions) in SBS format sitting drop plates via automation (Douglas Instruments Ltd) with 60 μl reagent reservoirs and drops composed of 0.2 μl protein with 0.2 μl reservoir. DH501 UCA was crystallized over a reservoir of 0.095 M sodium citrate pH 5.6, 20% isopropanol, and 20% PEG 4,000. All crystals were briefly soaked in reservoir supplemented with 20% ethylene glycol. Crystals were then cryocooled in liquid nitrogen.

Diffraction data for all crystals were collected at SER-CAT with an incident beam of 1 Å in wavelength. Data were reduced in HKL-2000 [77]. The DH501 UCA Fab structure was phased by molecular replacement in PHENIX [78] using the DH501 Fab structure separated into Fv and constant domains as search models [37].

For the DH501 UCA structure, rebuilding and real-space refinements were performed in Coot [79] with reciprocal space refinements in PHENIX [80] and validations in MolProbity [81]. It was noted that the average B factor for protein atoms (69.71 Å^2^; Table 1) was higher than that for solvent atoms (50.66 Å^2^), an inversion of what is observed for typical crystal structures. In fact the entire paratope of the DH501 UCA Fab showed elevated B factors. This could be due to an intrinsic mobility of the CDRs in the UCA antibody as noted in the results section however, it may also be a consequence of packing in the crystal lattice. Specifically, the paratope of the Fab was oriented toward a large solvent channel, thus lacking any potentially stabilizing crystal contacts. In support, the crystal structure of the mature DH501 had two Fab molecules in its asymmetric unit; one had similarly elevated B factors about residues in its paratope that were oriented toward a solvent channel but not in the same residues that were stabilized by crystal contacts in the other Fab [37]. Moreover, the solvent content of the mature DH501 lattice was modestly lower than that of the UCA– 46% compared to 50%, albeit it differently distributed throughout the crystals. The coordinates and structure factors for DH501 UCA unliganded Fab are deposited in the Protein Data Bank (PDB: 6P3B). Figures were generated in PyMol [41] with electrostatic potential surface calculations performed using the CHARMM solver [82].

**Table 1:** Crystallographic data collection and model refinement statistics.

Space group	C 1 2 1
Unit cell parameters *a*, *b*, *c* (Å)	80.18, 71.82, 87.69
Unit cell parameters *α*, *β*, *γ* (°)	90, 110.09, 90
Resolution (Å)	50.0–2.02 (2.05–2.02)^1^
No. of reflections collected	110034
No. of unique reflections	29777 (1161)
CC1/2	0.968 (0.838)
CC*	0.992 (0.955)
Mean I/σI	15.36 (1.71)
Rpim	0.063 (0.321)
Completeness (%)	96.2 (75.5)
Redundancy	3.7 (2.0)
*R*_work/_ *R*_free_ (%)	21.84 / 27.19 (32.16 / 35.72)
Number of protein atoms (non-hydrogen)	3210
Number of solvent atoms (non-hydrogen)	100
Average B value for protein atoms (Å^2^)	69.71
Average B value for solvent atoms (Å^2^)	50.66
RMSD bond lengths (Å)	0.003
RMSD bond angles (º)	0.690
Clashscore	6.59
Ramachandran favored (%)	91.9
Ramachandran outliers (%)	1.7

^1^Values in parentheses correspond to the highest resolution shell.

### Recombinant IgG production

Antibody was produced as previously described [37, 83]. Briefly, recombinant IgG1 were expressed in Expi293 cells (Invitrogen) by transient transfection with Expifectamine (Invitrogen). Five days after transfection cell culture media was cleared of cells by centrifugation and 0.8 μm filtration. IgG1 was purified from cell culture supernatant with protein A (ThermoFisher) affinity chromatography. Purified protein was buffer exchanged into PBS with successive rounds of centrifugation, filtered, and stored at -80°C.

### Differential scanning calorimetry

Antibody thermal denaturation profiles were determined as previously described [84]. Antibody profiles were generated in HEPES Buffered Saline (HBS; 10 mM HEPES, 150 mM NaCl pH 7.4) at concentrations ranging from 0.2–0.4 mg/mL using the NanoDSC platform (TA instruments; New Castle, DE). The observed, irreversible denaturation profiles were buffer subtracted, converted to molar heat capacity, baseline corrected with a 6^th^-order polynomial, and fit with three Gaussian transition models using the NanoAnalyze software (TA Instruments). The primary transition temperature (T_m_) is reported as the temperature at the maximum observed heat capacity.

### *In vitro* HIV-1 neutralization

Antibody-mediated HIV-1 neutralization was measured using Tat-regulated luciferase (Luc) reporter gene expression in TZM-bl cells as described previously [85]. TZM-bl cells were obtained from the NIH AIDS Research and Reference Reagent Program, as contributed by John Kappes and Xiaoyun Wu. Pseudoviruses were produced by transient transfection of 293T cells. To enrich for Man_9_GlcNAc_2_ glycosylation on HIV-1 envelope, the 293T producer cells were cultured in the presence of 25 μM kifunensine [42, 43]. The monoclonal antibody was pre-incubated with virus (~150,000 relative light unit equivalents) for 1 h at 37°C, and TZM-bl cells were subsequently added. After 48 h, cells were lysed and Luc activity determined with BriteLite Plus Reagent (Perkin Elmer) and a microtiter plate luminometer. Neutralization titers are the inhibitory concentration at which relative luminescence units (RLU) were reduced by 50% or 80% compared to RLU in virus only control wells after subtraction of background RLU in uninfected cell only control wells (IC50 and IC80 respectively).

### Multiplex oligomannose microsphere immunoassay

Oligomannose binding immunoassays were performed as described elsewhere [37]. Briefly, Lumavidin microspheres (Luminex) were coupled with 16 μM of biotinylated glycan. Biotinylated glycans were synthesized as described previously [37]. To reduce non-specific binding, Lumavidin microspheres were blocked with 3% BSA prior to coupling to glycans. Lumavidin microspheres were washed 3 times with 1% BSA in PBS, sonicated for 20 s, and vortexed for 20 s. The Lumavidin microspheres were mixed with 16 μM glycan in a final volume of 250 μL for 2 h at 2000 rpm at room temperature covered from light. Uncoupled glycan was washed away with 3 washes of 3% BSA in PBS and resuspended in 250 μL of PBS-BN (1% BSA, 0.05% NaN_3_ in PBS). The beads were sonicated for 20 s and vortexed for 20 s and were counted on a hemocytometer. Each bead set was diluted to the same Lumavidin microspheres concentration. For short-term storage the beads were kept at 4°C. For storage greater than 1 week the beads were stored at -80°C for up to 6 weeks.

Antibody binding was determined in 96-well filter plates (Millipore). 200 μg/mL of antibody was mixed with 0.5 μL of each Lumavidin microsphere in BSA-PBS-T (1% BSA, 0.05% Tween-20 in PBS). The antibodies were incubated in the dark with the microspheres for 1.5 h while shaking at 60 rpm on a microtiter plate shaker. The HIV-1 antibody 19B and anti-influenza antibody CH65 were used as negative controls. PGT128 and ConA were used as a positive control. Lumavidin microspheres were washed five times and antibody bound to glycan-coupled microsphere was detected with 100 μL of 2 μg/mL mouse anti-IgG-PE (Southern Biotech) or anti-ConA-PE for 1h. The secondary antibody was washed away with 5 washes of BSA-PBS-T, and binding of antibody to glycan was determined with a Bio-Plex 200 HTS (Bio-Rad) and Bioplex manager software (Bio-Rad). The values were represented as fluorescence minus fluorescence in wells with beads coupled with no glycan. Positive glycan binding was determined as values greater than 1800 and three-fold greater than values for anti-influenza antibody CH65 and linear HIV-1 peptide-reactive antibody 19B. 1800 was chosen as the lower bound for positivity based on the mean values from the negative controls over time.

### Site-directed mutagenesis

Single mutations were introduced into the DH501 heavy chain gene using the QuikChange Lightning Multi Site-Directed Mutagenesis Kit (Agilent). Oligonucleotides were synthesized and purified by standard desalting (IDT). Fifty nanograms of purified DNA was used as the template for mutagenesis and reactions were conducted per the manufacturer’s instructions except the volume of DpnI was doubled, and the DpnI digestion was conducted for 1 h.

### Recombinant HIV-1 gp140 SOSIP production

SOSIP gp140s were expressed, purified and characterized as previously described with minor modifications [83]. The BG505 SOSIP was designed as stated previously [86], and the design of the CON-S SOSIP gp140 will be described in a separate study. SOSIP gp140 was expressed in Freestyle293 cells (Invitrogen). On the day of transfection, Freestyle293 cells were diluted to 1.25x10^6^ cells/mL with fresh Freestyle293 media (Invitrogen) up to 1L total volume. The cells were co-transfected with plasmid DNA complexed with 293Fectin (Invitrogen). For each 1L transfection, 650 μg of SOSIP expressing plasmid and 150 μg of furin expressing plasmid were used. On day 6, cell culture supernatants were harvested by centrifugation of the cell culture for 30 min at 3500 rpm. The cell-free supernatant was filtered through a 0.8 μm filter and concentrated to less than 100 mL with a single-use tangential flow filtration cassettes and 0.8 μm filtered again. SOSIP protein was purified with PGT145 affinity chromatography. One hundred mg of PGT145 IgG1 antibody was conjugated to 10 mL of CnBr-activated Sepharose FastFlow resin (GE Healthcare). Coupled resin was packed into a Tricorn column (GE Healthcare), and stored in PBS plus 0.05% sodium azide. Cell-free supernatant was applied to the column at 2 mL/min using an AKTA Pure (GE Healthcare), washed, and protein was eluted off the column with 3M MgCl_2_. The eluate was immediately diluted in 10 mM Tris pH 8, 0.2 μm filtered, and concentrated down to 2 mL for size exclusion chromatography. Size exclusion chromatography was performed with a Superose6 16/600 column (GE Healthcare) in 10 mM Tris pH 8, 500 mM NaCl. Fractions containing trimeric HIV-1 Env protein were pooled together, sterile-filtered, snap frozen, and stored at -80°C. The formation of trimers was determined by negative stain electron microscopy, blue native-PAGE, and analytical size exclusion chromatography.

### Surface plasmon resonance (SPR)

SPR experiments were performed on a BIACore T200 as described in detail previously [37, 84]. Approximately 300 RU (range 310–321 RU) of each antibody was captured on an anti-human IgFc immobilized Series S CM5 sensor chip (GE Healthcare). SOSIP Env was flowed over immobilized antibody for 200 s in HEPES buffered saline, and dissociation was measured for 200 s. In between injections of each Env, the surface was regenerated by injecting glycine pH2 for 30 s. Curve fitting analysis was performed with BiaEvaluation software (GE Healthcare) using a 1:1 Langmuir model or heterogeneous ligand depending on the curve fit. Background binding was determined as binding signal obtained for the anti-influenza virus antibody CH65 binding to SOSIP gp140. Background binding was subtracted from the values obtained for each DH501 antibody variant. Spikes in binding responses when analyte flow was stopped or started were removed.

### Statistical analyses

Statistical comparisons of antibody functions or antibody characteristics were not performed because the sample size is too small for parametric testing (i.e., t-test) and nonparametric testing (i.e., an exact Wilcoxon test based on the ranked values) could lead to statistically significant findings that are not biologically relevant. Therefore, only descriptive statistics are provided based on calculations from Prism v8.0 (Graphpad).

## Supporting information

S1 Fig Glycan binding of anti-influenza antibody CH65 and HIV-1 linear peptide antibody 19B. Both antibodies serve as negative controls in each glycan binding assay. Mean binding values observed for CH65 and 19B are considered background and positivity thresholds are set to be 3-fold above the background binding value. Representative graphs are shown for each control antibody or the positive control lectin ConA.
(TIF)

S2 Fig Membrane bound DH501 IgG binds Man_9_GlcNAc_2_-glycosylated HIV-1 peptide, but DH501 UCA does not. Flow cytometric analysis of DH501 UCA IgG and DH501 IgG (**A**) cell surface expression, (**B**) Man_9_GlcNAc_2_-glycosylated peptide binding, and (**C**) aglycone peptide binding. Mock indicates cells transfected without DNA encoding an antibody. Bar graphs show the mean percentage of positive cells from two independent experiments.
(TIF)

S3 Fig DH501 with a minimal number of somatic mutations lack detectable glycan reactivity. (**A**) Amino acid alignment of the V_H_ of DH501 and minimally somatically-mutated DH501 variants (DH501.min1-4). The set of amino acids in green and red were added to DH270.min1 individually or together to generate DH270.min2-4 (**B**) Binding of DH501 and DH501.min variants to Man_7_GlcNAc_2_ D1 (7), Man_8_GlcNAc_2_ D1D3 (8a), Man_8_GlcNAc_2_ D1D2 (8b), Man_9_GlcNAc_2_ (9). Mean and standard error are shown for triplicate experiments. Positive glycan binding based on negative control antibody binding is shown as a filled bar. Open bars indicate negative binding values. Positivity thresholds for 7, 8a, 8b, and 9 are 0.2x10^4^, 0.15x10^4^, 0.15x10^4^, 0.2x10^4^ respectively.
(TIF)

## References

[1] Rey FA, Lok SM. Common Features of Enveloped Viruses and Implications for Immunogen Design for Next-Generation Vaccines. Cell. 2018;172(6):1319–34. Epub 2018/03/10. 10.1016/j.cell.2018.02.054 . 29522750 PMC7112304

[2] Vigerust DJ, Shepherd VL. Virus glycosylation: role in virulence and immune interactions. Trends Microbiol. 2007;15(5):211–8. Epub 2007/04/03. 10.1016/j.tim.2007.03.003 . 17398101 PMC7127133

[3] Helle F, Vieyres G, Elkrief L, Popescu CI, Wychowski C, Descamps V, et al Role of N-linked glycans in the functions of hepatitis C virus envelope proteins incorporated into infectious virions. Journal of virology. 2010;84(22):11905–15. Epub 2010/09/17. 10.1128/JVI.01548-10 20844034 PMC2977866

[4] Falkowska E, Kajumo F, Garcia E, Reinus J, Dragic T. Hepatitis C virus envelope glycoprotein E2 glycans modulate entry, CD81 binding, and neutralization. Journal of virology. 2007;81(15):8072–9. Epub 2007/05/18. 10.1128/JVI.00459-07 17507469 PMC1951298

[5] Moldt B, Rakasz EG, Schultz N, Chan-Hui PY, Swiderek K, Weisgrau KL, et al Highly potent HIV-specific antibody neutralization in vitro translates into effective protection against mucosal SHIV challenge in vivo. Proceedings of the National Academy of Sciences of the United States of America. 2012;109(46):18921–5. Epub 2012/10/27. 10.1073/pnas.1214785109 23100539 PMC3503218

[6] Hessell AJ, Rakasz EG, Poignard P, Hangartner L, Landucci G, Forthal DN, et al Broadly neutralizing human anti-HIV antibody 2G12 is effective in protection against mucosal SHIV challenge even at low serum neutralizing titers. PLoS pathogens. 2009;5(5):e1000433 Epub 2009/05/14. 10.1371/journal.ppat.1000433 19436712 PMC2674935

[7] Lopez-Ribot JL, Casanova M, Murgui A, Martinez JP. Antibody response to Candida albicans cell wall antigens. FEMS Immunol Med Microbiol. 2004;41(3):187–96. Epub 2004/06/16. 10.1016/j.femsim.2004.03.012 . 15196567

[8] Wei X, Decker JM, Wang S, Hui H, Kappes JC, Wu X, et al Antibody neutralization and escape by HIV-1. Nature. 2003;422(6929):307–12. Epub 2003/03/21. 10.1038/nature01470 . 12646921

[9] Seymour RM, Allan MJ, Pomiankowski A, Gustafsson K. Evolution of the human ABO polymorphism by two complementary selective pressures. Proc Biol Sci. 2004;271(1543):1065–72. Epub 2004/08/06. 10.1098/rspb.2004.2674 15293861 PMC1691687

[10] Scanlan CN, Offer J, Zitzmann N, Dwek RA. Exploiting the defensive sugars of HIV-1 for drug and vaccine design. Nature. 2007;446(7139):1038–45. Epub 2007/04/27. 10.1038/nature05818 . 17460665

[11] Leonard CK, Spellman MW, Riddle L, Harris RJ, Thomas JN, Gregory TJ. Assignment of intrachain disulfide bonds and characterization of potential glycosylation sites of the type 1 recombinant human immunodeficiency virus envelope glycoprotein (gp120) expressed in Chinese hamster ovary cells. J Biol Chem. 1990;265(18):10373–82. Epub 1990/06/25. . 2355006

[12] Astronomo RD, Burton DR. Carbohydrate vaccines: developing sweet solutions to sticky situations? Nat Rev Drug Discov. 2010;9(4):308–24. Epub 2010/04/02. 10.1038/nrd3012 20357803 PMC3878310

[13] Go EP, Irungu J, Zhang Y, Dalpathado DS, Liao HX, Sutherland LL, et al Glycosylation site-specific analysis of HIV envelope proteins (JR-FL and CON-S) reveals major differences in glycosylation site occupancy, glycoform profiles, and antigenic epitopes' accessibility. J Proteome Res. 2008;7(4):1660–74. Epub 2008/03/12. 10.1021/pr7006957 18330979 PMC3658474

[14] Mizuochi T, Matthews TJ, Kato M, Hamako J, Titani K, Solomon J, et al Diversity of oligosaccharide structures on the envelope glycoprotein gp 120 of human immunodeficiency virus 1 from the lymphoblastoid cell line H9. Presence of complex-type oligosaccharides with bisecting N-acetylglucosamine residues. J Biol Chem. 1990;265(15):8519–24. Epub 1990/05/25. . 2341393

[15] Go EP, Chang Q, Liao HX, Sutherland LL, Alam SM, Haynes BF, et al Glycosylation site-specific analysis of clade C HIV-1 envelope proteins. J Proteome Res. 2009;8(9):4231–42. Epub 2009/07/21. 10.1021/pr9002728 19610667 PMC2756219

[16] Go EP, Herschhorn A, Gu C, Castillo-Menendez L, Zhang S, Mao Y, et al Comparative Analysis of the Glycosylation Profiles of Membrane-Anchored HIV-1 Envelope Glycoprotein Trimers and Soluble gp140. Journal of virology. 2015;89(16):8245–57. Epub 2015/05/29. 10.1128/JVI.00628-15 26018173 PMC4524223

[17] Behrens AJ, Vasiljevic S, Pritchard LK, Harvey DJ, Andev RS, Krumm SA, et al Composition and Antigenic Effects of Individual Glycan Sites of a Trimeric HIV-1 Envelope Glycoprotein. Cell reports. 2016;14(11):2695–706. Epub 2016/03/15. 10.1016/j.celrep.2016.02.058 26972002 PMC4805854

[18] Doria-Rose NA, Altae-Tran HR, Roark RS, Schmidt SD, Sutton MS, Louder MK, et al Mapping Polyclonal HIV-1 Antibody Responses via Next-Generation Neutralization Fingerprinting. PLoS pathogens. 2017;13(1):e1006148 Epub 2017/01/05. 10.1371/journal.ppat.1006148 28052137 PMC5241146

[19] Walker LM, Simek MD, Priddy F, Gach JS, Wagner D, Zwick MB, et al A limited number of antibody specificities mediate broad and potent serum neutralization in selected HIV-1 infected individuals. PLoS pathogens. 2010;6(8):e1001028 10.1371/journal.ppat.1001028 20700449 PMC2916884

[20] Walker LM, Huber M, Doores KJ, Falkowska E, Pejchal R, Julien JP, et al Broad neutralization coverage of HIV by multiple highly potent antibodies. Nature. 2011;477(7365):466–70. Epub 2011/08/19. 10.1038/nature10373 21849977 PMC3393110

[21] Walker LM, Phogat SK, Chan-Hui PY, Wagner D, Phung P, Goss JL, et al Broad and potent neutralizing antibodies from an African donor reveal a new HIV-1 vaccine target. Science. 2009;326(5950):285–9. Epub 2009/09/05. 10.1126/science.1178746 19729618 PMC3335270

[22] Bonsignori M, Kreider EF, Fera D, Meyerhoff RR, Bradley T, Wiehe K, et al Staged induction of HIV-1 glycan-dependent broadly neutralizing antibodies. Sci Transl Med. 2017;9(381). Epub 2017/03/17. 10.1126/scitranslmed.aai7514 28298420 PMC5562350

[23] Bonsignori M, Montefiori DC, Wu X, Chen X, Hwang KK, Tsao CY, et al Two distinct broadly neutralizing antibody specificities of different clonal lineages in a single HIV-1-infected donor: implications for vaccine design. J Virol. 2012;86(8):4688–92. Epub 2012/02/04. 10.1128/JVI.07163-11 22301150 PMC3318651

[24] Kong L, Lee JH, Doores KJ, Murin CD, Julien JP, McBride R, et al Supersite of immune vulnerability on the glycosylated face of HIV-1 envelope glycoprotein gp120. Nature structural & molecular biology. 2013;20(7):796–803. Epub 2013/05/28. 10.1038/nsmb.2594 23708606 PMC3823233

[25] Barnes CO, Gristick HB, Freund NT, Escolano A, Lyubimov AY, Hartweger H, et al Structural characterization of a highly-potent V3-glycan broadly neutralizing antibody bound to natively-glycosylated HIV-1 envelope. Nature communications. 2018;9(1):1251 Epub 2018/03/30. 10.1038/s41467-018-03632-y 29593217 PMC5871869

[26] Kong L, Torrents de la Pena A, Deller MC, Garces F, Sliepen K, Hua Y, et al Complete epitopes for vaccine design derived from a crystal structure of the broadly neutralizing antibodies PGT128 and 8ANC195 in complex with an HIV-1 Env trimer. Acta crystallographica Section D, Biological crystallography. 2015;71(Pt 10):2099–108. 10.1107/S1399004715013917 26457433 PMC4601371

[27] Pejchal R, Doores KJ, Walker LM, Khayat R, Huang PS, Wang SK, et al A potent and broad neutralizing antibody recognizes and penetrates the HIV glycan shield. Science. 2011;334(6059):1097–103. 10.1126/science.1213256 21998254 PMC3280215

[28] Calarese DA, Scanlan CN, Zwick MB, Deechongkit S, Mimura Y, Kunert R, et al Antibody domain exchange is an immunological solution to carbohydrate cluster recognition. Science. 2003;300(5628):2065–71. Epub 2003/06/28. 10.1126/science.1083182 . 12829775

[29] Murin CD, Julien JP, Sok D, Stanfield RL, Khayat R, Cupo A, et al Structure of 2G12 Fab2 in complex with soluble and fully glycosylated HIV-1 Env by negative-stain single-particle electron microscopy. Journal of virology. 2014;88(17):10177–88. Epub 2014/06/27. 10.1128/JVI.01229-14 24965454 PMC4136306

[30] Pegu A, Yang ZY, Boyington JC, Wu L, Ko SY, Schmidt SD, et al Neutralizing antibodies to HIV-1 envelope protect more effectively in vivo than those to the CD4 receptor. Science translational medicine. 2014;6(243):243ra88 Epub 2014/07/06. 10.1126/scitranslmed.3008992 24990883 PMC4562469

[31] Gautam R, Nishimura Y, Pegu A, Nason MC, Klein F, Gazumyan A, et al A single injection of anti-HIV-1 antibodies protects against repeated SHIV challenges. Nature. 2016;533(7601):105–9. Epub 2016/04/28. 10.1038/nature17677 27120156 PMC5127204

[32] Campbell CT, Llewellyn SR, Demberg T, Morgan IL, Robert-Guroff M, Gildersleeve JC. High-throughput profiling of anti-glycan humoral responses to SIV vaccination and challenge. PloS one. 2013;8(9):e75302 Epub 2013/10/03. 10.1371/journal.pone.0075302 24086502 PMC3781036

[33] Pantophlet R, Trattnig N, Murrell S, Lu N, Chau D, Rempel C, et al Bacterially derived synthetic mimetics of mammalian oligomannose prime antibody responses that neutralize HIV infectivity. Nature communications. 2017;8(1):1601 Epub 2017/11/19. 10.1038/s41467-017-01640-y 29150603 PMC5693931

[34] Luallen RJ, Lin J, Fu H, Cai KK, Agrawal C, Mboudjeka I, et al An engineered Saccharomyces cerevisiae strain binds the broadly neutralizing human immunodeficiency virus type 1 antibody 2G12 and elicits mannose-specific gp120-binding antibodies. Journal of virology. 2008;82(13):6447–57. Epub 2008/04/25. 10.1128/JVI.00412-08 18434410 PMC2447081

[35] Doores KJ, Fulton Z, Hong V, Patel MK, Scanlan CN, Wormald MR, et al A nonself sugar mimic of the HIV glycan shield shows enhanced antigenicity. Proceedings of the National Academy of Sciences of the United States of America. 2010;107(40):17107–12. Epub 2010/09/21. 10.1073/pnas.1002717107 20852065 PMC2951454

[36] Astronomo RD, Lee HK, Scanlan CN, Pantophlet R, Huang CY, Wilson IA, et al A glycoconjugate antigen based on the recognition motif of a broadly neutralizing human immunodeficiency virus antibody, 2G12, is immunogenic but elicits antibodies unable to bind to the self glycans of gp120. Journal of virology. 2008;82(13):6359–68. Epub 2008/04/25. 10.1128/JVI.00293-08 18434393 PMC2447108

[37] Saunders KO, Nicely NI, Wiehe K, Bonsignori M, Meyerhoff RR, Parks R, et al Vaccine Elicitation of High Mannose-Dependent Neutralizing Antibodies against the V3-Glycan Broadly Neutralizing Epitope in Nonhuman Primates. Cell reports. 2017;18(9):2175–88. 10.1016/j.celrep.2017.02.003 28249163 PMC5408352

[38] Scanlan CN, Ritchie GE, Baruah K, Crispin M, Harvey DJ, Singer BB, et al Inhibition of mammalian glycan biosynthesis produces non-self antigens for a broadly neutralising, HIV-1 specific antibody. J Mol Biol. 2007;372(1):16–22. Epub 2007 Jun 16. 10.1016/j.jmb.2007.06.027 17631311

[39] Bonomelli C, Doores KJ, Dunlop DC, Thaney V, Dwek RA, Burton DR, et al The glycan shield of HIV is predominantly oligomannose independently of production system or viral clade. PloS one. 2011;6(8):e23521 Epub 2011/08/23. 10.1371/journal.pone.0023521 21858152 PMC3156772

[40] Doores KJ, Bonomelli C, Harvey DJ, Vasiljevic S, Dwek RA, Burton DR, et al Envelope glycans of immunodeficiency virions are almost entirely oligomannose antigens. Proceedings of the National Academy of Sciences of the United States of America. 2010;107(31):13800–5. Epub 2010/07/21. 10.1073/pnas.1006498107 20643940 PMC2922250

[41] Schrödinger L. The PyMOL Molecular Graphics System 2.0 ed.

[42] Scanlan CN, Ritchie GE, Baruah K, Crispin M, Harvey DJ, Singer BB, et al Inhibition of mammalian glycan biosynthesis produces non-self antigens for a broadly neutralising, HIV-1 specific antibody. J Mol Biol. 2007;372(1):16–22. Epub 2007/07/17. 10.1016/j.jmb.2007.06.027 . 17631311

[43] Doores KJ, Burton DR. Variable loop glycan dependency of the broad and potent HIV-1-neutralizing antibodies PG9 and PG16. Journal of virology. 2010;84(20):10510–21. 10.1128/JVI.00552-10 20686044 PMC2950566

[44] Kepler TB. Reconstructing a B-cell clonal lineage. I. Statistical inference of unobserved ancestors. F1000Res. 2013;2:103 Epub 2014/02/21. 10.12688/f1000research.2-103.v1 24555054 PMC3901458

[45] Jones PT, Dear PH, Foote J, Neuberger MS, Winter G. Replacing the complementarity-determining regions in a human antibody with those from a mouse. Nature. 1986;321(6069):522–5. Epub 1986/05/04. 10.1038/321522a0 . 3713831

[46] Georgiev IS, Rudicell RS, Saunders KO, Shi W, Kirys T, McKee K, et al Antibodies VRC01 and 10E8 neutralize HIV-1 with high breadth and potency even with Ig-framework regions substantially reverted to germline. J Immunol. 2014;192(3):1100–6. Epub 2014/01/07. 10.4049/jimmunol.1302515 24391217 PMC4140862

[47] Jardine JG, Sok D, Julien JP, Briney B, Sarkar A, Liang CH, et al Minimally Mutated HIV-1 Broadly Neutralizing Antibodies to Guide Reductionist Vaccine Design. PLoS pathogens. 2016;12(8):e1005815 Epub 2016/08/26. 10.1371/journal.ppat.1005815 27560183 PMC4999182

[48] Klein F, Diskin R, Scheid JF, Gaebler C, Mouquet H, Georgiev IS, et al Somatic mutations of the immunoglobulin framework are generally required for broad and potent HIV-1 neutralization. Cell. 2013;153(1):126–38. Epub 2013/04/02. 10.1016/j.cell.2013.03.018 23540694 PMC3792590

[49] Zhou T, Georgiev I, Wu X, Yang ZY, Dai K, Finzi A, et al Structural basis for broad and potent neutralization of HIV-1 by antibody VRC01. Science. 2010;329(5993):811–7. Epub 2010/07/10. 10.1126/science.1192819 20616231 PMC2981354

[50] Li SC, Goto NK, Williams KA, Deber CM. Alpha-helical, but not beta-sheet, propensity of proline is determined by peptide environment. Proceedings of the National Academy of Sciences of the United States of America. 1996;93(13):6676–81. Epub 1996/06/25. 10.1073/pnas.93.13.6676 8692877 PMC39085

[51] von Heijne G. Proline kinks in transmembrane alpha-helices. J Mol Biol. 1991;218(3):499–503. Epub 1991/04/05. 10.1016/0022-2836(91)90695-3 . 2016741

[52] Vermeer AW, Norde W. The thermal stability of immunoglobulin: unfolding and aggregation of a multi-domain protein. Biophys J. 2000;78(1):394–404. Epub 2000/01/05. 10.1016/S0006-3495(00)76602-1 10620303 PMC1300647

[53] Goto Y, Ichimura N, Hamaguchi K. Effects of ammonium sulfate on the unfolding and refolding of the variable and constant fragments of an immunoglobulin light chain. Biochemistry. 1988;27(5):1670–7. Epub 1988/03/08. 10.1021/bi00405a043 . 3130099

[54] Lee JH, Andrabi R, Su CY, Yasmeen A, Julien JP, Kong L, et al A Broadly Neutralizing Antibody Targets the Dynamic HIV Envelope Trimer Apex via a Long, Rigidified, and Anionic beta-Hairpin Structure. Immunity. 2017;46(4):690–702. 10.1016/j.immuni.2017.03.017 28423342 PMC5400778

[55] McLellan JS, Pancera M, Carrico C, Gorman J, Julien JP, Khayat R, et al Structure of HIV-1 gp120 V1/V2 domain with broadly neutralizing antibody PG9. Nature. 2011;480(7377):336–43. 10.1038/nature10696 22113616 PMC3406929

[56] Garces F, Sok D, Kong L, McBride R, Kim HJ, Saye-Francisco KF, et al Structural evolution of glycan recognition by a family of potent HIV antibodies. Cell. 2014;159(1):69–79. Epub 2014/09/27. 10.1016/j.cell.2014.09.009 25259921 PMC4278586

[57] Doores KJ, Kong L, Krumm SA, Le KM, Sok D, Laserson U, et al Two classes of broadly neutralizing antibodies within a single lineage directed to the high-mannose patch of HIV envelope. Journal of virology. 2015;89(2):1105–18. 10.1128/JVI.02905-14 25378488 PMC4300629

[58] Mouquet H, Scharf L, Euler Z, Liu Y, Eden C, Scheid JF, et al Complex-type N-glycan recognition by potent broadly neutralizing HIV antibodies. Proceedings of the National Academy of Sciences of the United States of America. 2012;109(47):E3268–77. 10.1073/pnas.1217207109 23115339 PMC3511153

[59] Lavie M, Hanoulle X, Dubuisson J. Glycan Shielding and Modulation of Hepatitis C Virus Neutralizing Antibodies. Frontiers in immunology. 2018;9:910 Epub 2018/05/15. 10.3389/fimmu.2018.00910 29755477 PMC5934428

[60] West AP Jr., Diskin R, Nussenzweig MC, Bjorkman PJ. Structural basis for germ-line gene usage of a potent class of antibodies targeting the CD4-binding site of HIV-1 gp120. Proc Natl Acad Sci U S A. 2012;109(30):E2083–90. Epub 2012/06/30. 10.1073/pnas.1208984109 22745174 PMC3409792

[61] Corti D, Suguitan AL Jr., Pinna D, Silacci C, Fernandez-Rodriguez BM, Vanzetta F, et al Heterosubtypic neutralizing antibodies are produced by individuals immunized with a seasonal influenza vaccine. J Clin Invest. 2010;120(5):1663–73. Epub 2010/04/15. 10.1172/JCI41902 20389023 PMC2860935

[62] Wrammert J, Koutsonanos D, Li GM, Edupuganti S, Sui J, Morrissey M, et al Broadly cross-reactive antibodies dominate the human B cell response against 2009 pandemic H1N1 influenza virus infection. J Exp Med. 2011;208(1):181–93. Epub 2011/01/12. 10.1084/jem.20101352 21220454 PMC3023136

[63] Lingwood D, McTamney PM, Yassine HM, Whittle JR, Guo X, Boyington JC, et al Structural and genetic basis for development of broadly neutralizing influenza antibodies. Nature. 2012;489(7417):566–70. Epub 2012/08/31. 10.1038/nature11371 . 22932267 PMC7095019

[64] Bassing CH, Swat W, Alt FW. The mechanism and regulation of chromosomal V(D)J recombination. Cell. 2002;109 Suppl:S45–55. Epub 2002/05/02. 10.1016/s0092-8674(02)00675-x . 11983152

[65] Weigert M, Perry R, Kelley D, Hunkapiller T, Schilling J, Hood L. The joining of V and J gene segments creates antibody diversity. Nature. 1980;283(5746):497–9. Epub 1980/01/31. 10.1038/283497a0 6766210

[66] Brack C, Hirama M, Lenhard-Schuller R, Tonegawa S. A complete immunoglobulin gene is created by somatic recombination. Cell. 1978;15(1):1–14. Epub 1978/09/01. 10.1016/0092-8674(78)90078-8 100225

[67] Sakano H, Kurosawa Y, Weigert M, Tonegawa S. Identification and nucleotide sequence of a diversity DNA segment (D) of immunoglobulin heavy-chain genes. Nature. 1981;290(5807):562–5. Epub 1981/04/16. 10.1038/290562a0 . 6783961

[68] Early P, Huang H, Davis M, Calame K, Hood L. An immunoglobulin heavy chain variable region gene is generated from three segments of DNA: VH, D and JH. Cell. 1980;19(4):981–92. Epub 1980/04/01. 10.1016/0092-8674(80)90089-6 . 6769593

[69] Soto C, Bombardi RG, Branchizio A, Kose N, Matta P, Sevy AM, et al High frequency of shared clonotypes in human B cell receptor repertoires. Nature. 2019;566(7744):398–402. Epub 2019/02/15. 10.1038/s41586-019-0934-8 . 30760926 PMC6949180

[70] Willis JR, Finn JA, Briney B, Sapparapu G, Singh V, King H, et al Long antibody HCDR3s from HIV-naive donors presented on a PG9 neutralizing antibody background mediate HIV neutralization. Proceedings of the National Academy of Sciences of the United States of America. 2016;113(16):4446–51. 10.1073/pnas.1518405113 27044078 PMC4843476

[71] Briney B, Inderbitzin A, Joyce C, Burton DR. Commonality despite exceptional diversity in the baseline human antibody repertoire. Nature. 2019;566(7744):393–7. Epub 2019/01/22. 10.1038/s41586-019-0879-y 30664748 PMC6411386

[72] Amzel LM, Poljak RJ. Three-dimensional structure of immunoglobulins. Annu Rev Biochem. 1979;48:961–97. Epub 1979/01/01. 10.1146/annurev.bi.48.070179.004525 . 89832

[73] Porter RR. Structural studies of immunoglobulins. Science. 1973;180(4087):713–6. Epub 1973/05/18. 10.1126/science.180.4087.713 . 4122075

[74] Trevino SR, Schaefer S, Scholtz JM, Pace CN. Increasing protein conformational stability by optimizing beta-turn sequence. J Mol Biol. 2007;373(1):211–8. Epub 2007/09/04. 10.1016/j.jmb.2007.07.061 17765922 PMC2084202

[75] Nicely NI, Dennison SM, Spicer L, Scearce RM, Kelsoe G, Ueda Y, et al Crystal structure of a non-neutralizing antibody to the HIV-1 gp41 membrane-proximal external region. Nat Struct Mol Biol. 2010;17(12):1492–4. Epub 2010/11/16. 10.1038/nsmb.1944 . 21076400 PMC6081738

[76] Liao HX, Sutherland LL, Xia SM, Brock ME, Scearce RM, Vanleeuwen S, et al A group M consensus envelope glycoprotein induces antibodies that neutralize subsets of subtype B and C HIV-1 primary viruses. Virology. 2006;353(2):268–82. Epub 2006/10/14. 10.1016/j.virol.2006.04.043 17039602 PMC1762135

[77] Otwinowski Z, Minor W. Processing of X-ray diffraction data collected in oscillation mode. Methods Enzymol. 1997;276:307–26. Epub 1997/01/01. . 27754618 10.1016/S0076-6879(97)76066-X

[78] Terwilliger TC, Grosse-Kunstleve RW, Afonine PV, Moriarty NW, Zwart PH, Hung LW, et al Iterative model building, structure refinement and density modification with the PHENIX AutoBuild wizard. Acta Crystallogr D Biol Crystallogr. 2008;64(Pt 1):61–9. Epub 2007/12/21. 10.1107/S090744490705024X 18094468 PMC2394820

[79] Emsley P, Lohkamp B, Scott WG, Cowtan K. Features and development of Coot. Acta Crystallogr D Biol Crystallogr. 2010;66(Pt 4):486–501. Epub 2010/04/13. 10.1107/S0907444910007493 20383002 PMC2852313

[80] Adams PD, Afonine PV, Bunkoczi G, Chen VB, Davis IW, Echols N, et al PHENIX: a comprehensive Python-based system for macromolecular structure solution. Acta Crystallogr D Biol Crystallogr. 2010;66(Pt 2):213–21. Epub 2010/02/04. 10.1107/S0907444909052925 20124702 PMC2815670

[81] Lovell SC, Davis IW, Arendall WB 3rd, de Bakker PI, Word JM, Prisant MG, et al Structure validation by Calpha geometry: phi,psi and Cbeta deviation. Proteins. 2003;50(3):437–50. Epub 2003/01/31. 10.1002/prot.10286 . 12557186

[82] Jo S, Kim T, Iyer VG, Im W. CHARMM-GUI: A Web-based Graphical User Interface for CHARMM. J Comput Chem. 2008;29:1859–65. 10.1002/jcc.20945 18351591

[83] Saunders KO, Verkoczy LK, Jiang C, Zhang J, Parks R, Chen H, et al Vaccine Induction of Heterologous Tier 2 HIV-1 Neutralizing Antibodies in Animal Models. Cell reports. 2017;21(13):3681–90. Epub 2017/12/28. 10.1016/j.celrep.2017.12.028 PubMed Central PMCID: PMC5777169. 29281818 PMC5777169

[84] Henderson R, Watts BE, Ergin HN, Anasti K, Parks R, Xia SM, et al Selection of immunoglobulin elbow region mutations impacts interdomain conformational flexibility in HIV-1 broadly neutralizing antibodies. Nat Commun. 2019;10(1):654 Epub 2019/02/10. 10.1038/s41467-019-08415-7 30737386 PMC6368608

[85] Montefiori DC. Measuring HIV neutralization in a luciferase reporter gene assay. Methods Mol Biol. 2009;485:395–405. Epub 2008/11/21. 10.1007/978-1-59745-170-3_26 . 19020839

[86] Sanders RW, Derking R, Cupo A, Julien JP, Yasmeen A, de Val N, et al A next-generation cleaved, soluble HIV-1 Env trimer, BG505 SOSIP.664 gp140, expresses multiple epitopes for broadly neutralizing but not non-neutralizing antibodies. PLoS pathogens. 2013;9(9):e1003618 Epub 2013/09/27. 10.1371/journal.ppat.1003618 24068931 PMC3777863

