# Supplementary figures and images for: Cooperation between somatic mutation and germline-encoded residues enables antibody recognition of HIV-1 envelope glycans
Source: PLoS Pathog. 2019 Dec 16;15(12):e1008165. doi: 10.1371/journal.ppat.1008165 (PMC6936856; doi:10.1371/journal.ppat.1008165)

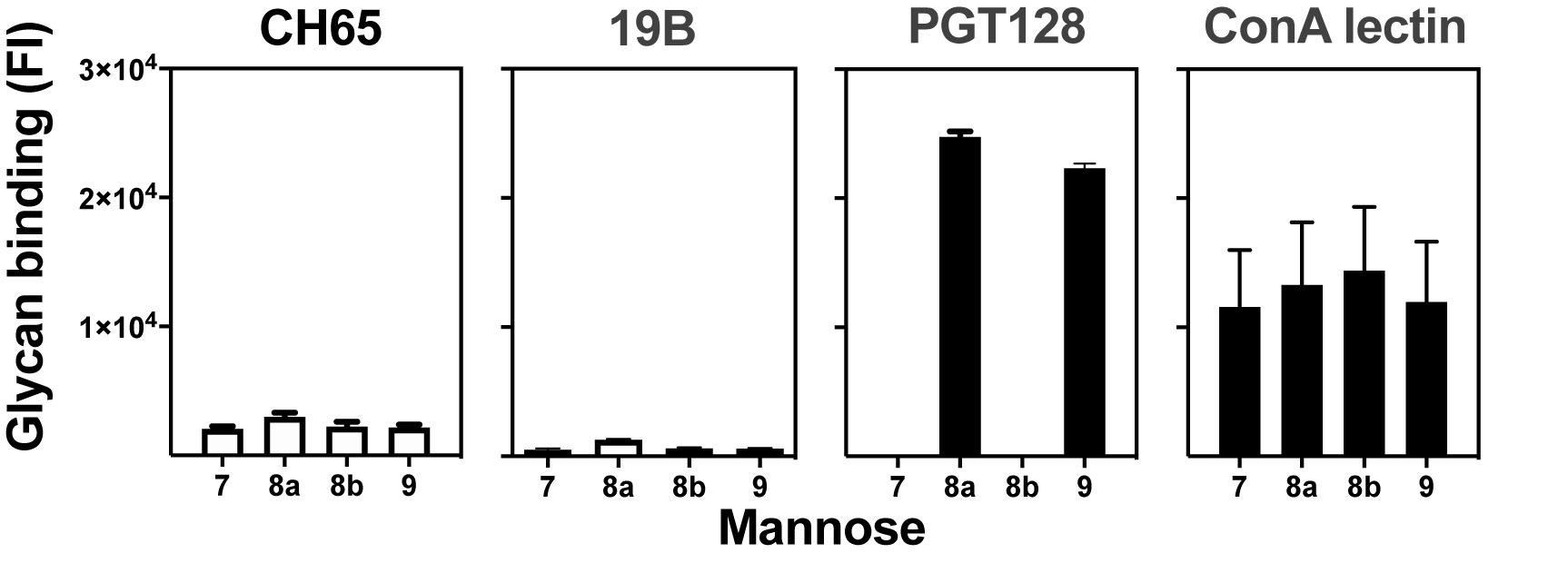

Supplement: S1 Fig — Both antibodies serve as negative controls in each glycan binding assay. Mean binding values observed for CH65 and 19B are considered background and positivity thresholds are set to be 3-fold above the background binding value. Representative graphs are shown for each control antibody or the positive control lectin ConA. (TIF) [file ppat.1008165.s001.tif]

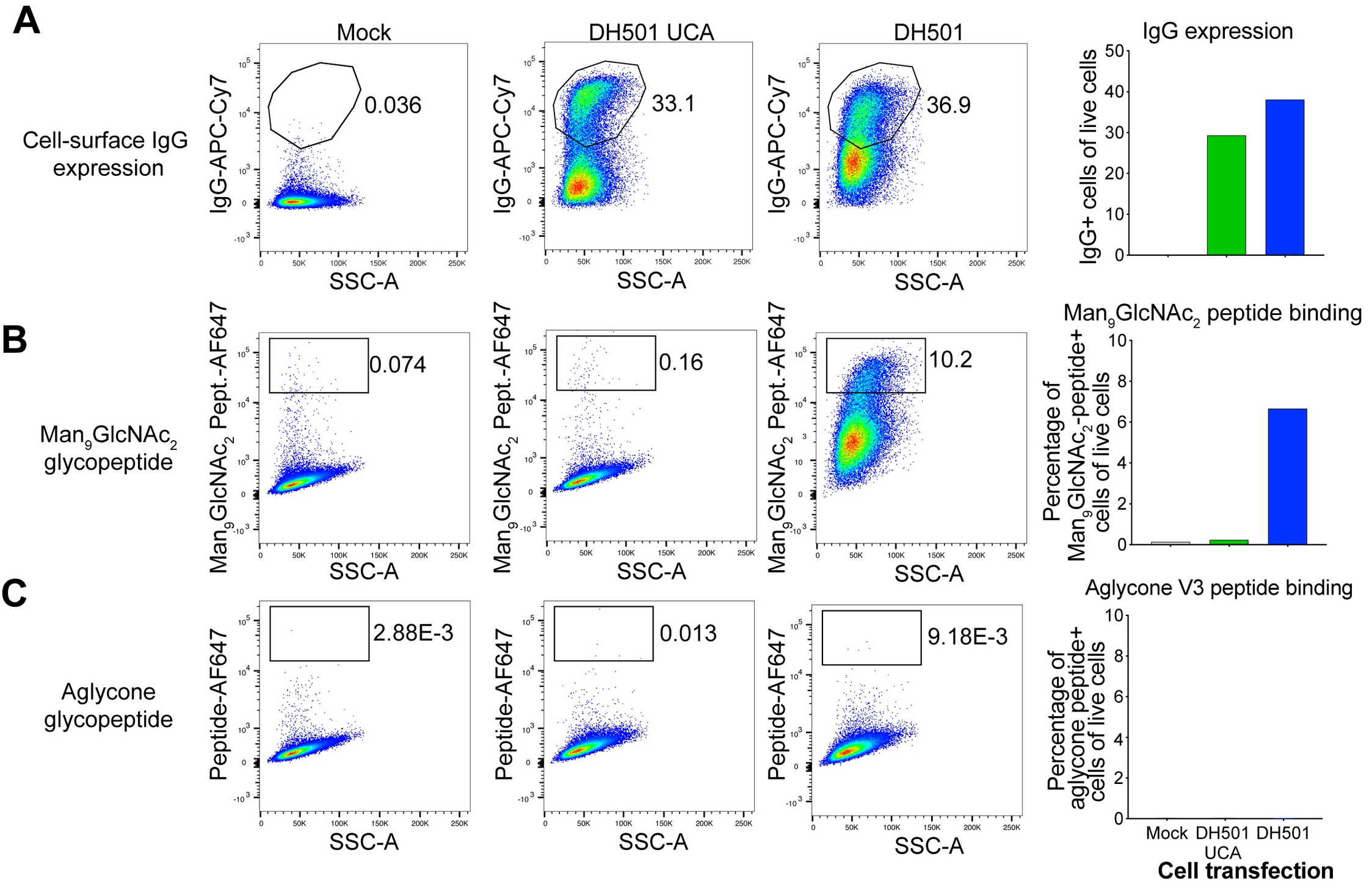

Supplement: S2 Fig — Flow cytometric analysis of DH501 UCA IgG and DH501 IgG (A) cell surface expression, (B) Man9GlcNAc2-glycosylated peptide binding, and (C) aglycone peptide binding. Mock indicates cells transfected without DNA encoding an antibody. Bar graphs show the mean percentage of positive cells from two independent experiments. (TIF) [file ppat.1008165.s002.tif]

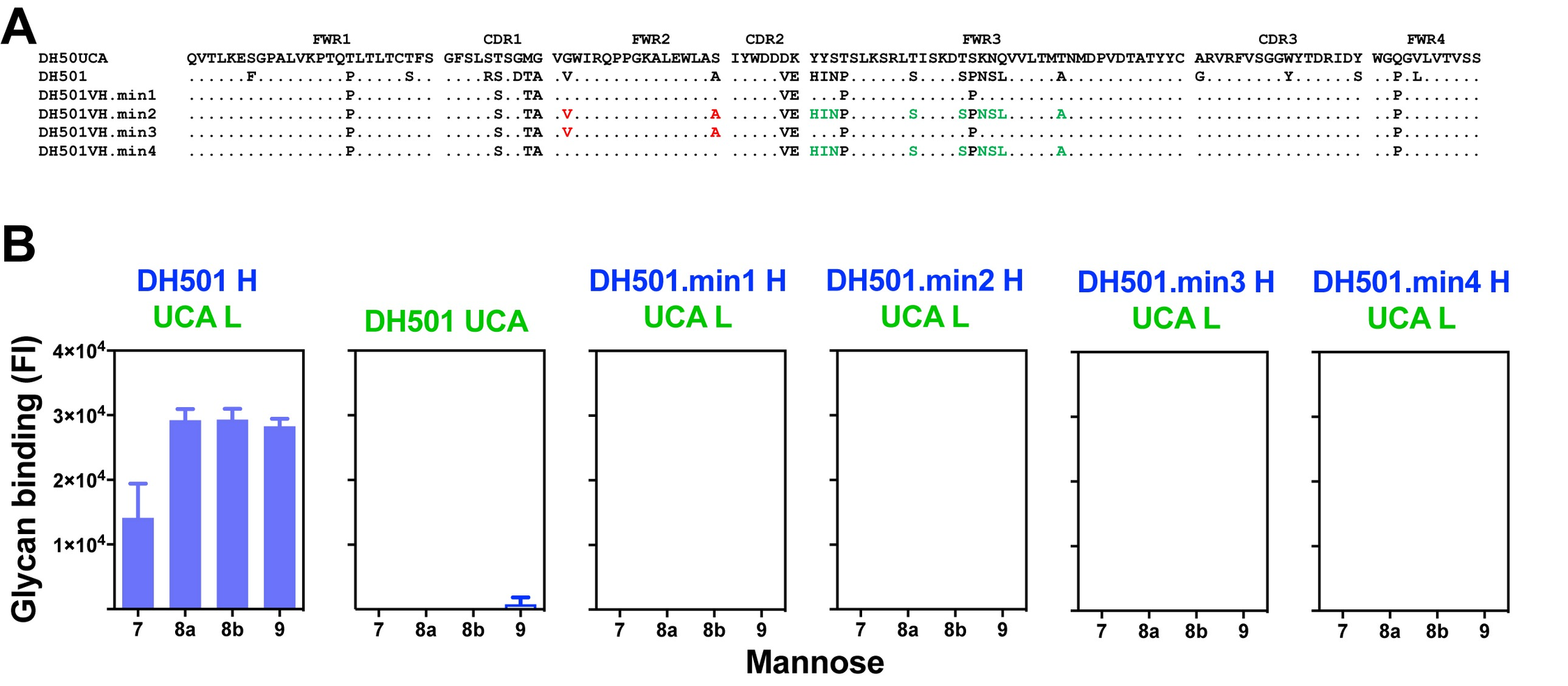

Supplement: S3 Fig — (A) Amino acid alignment of the VH of DH501 and minimally somatically-mutated DH501 variants (DH501.min1-4). The set of amino acids in green and red were added to DH270.min1 individually or together to generate DH270.min2-4 (B) Binding of DH501 and DH501.min variants to Man7GlcNAc2 D1 (7), Man8GlcNAc2 D1D3 (8a), Man8GlcNAc2 D1D2 (8b), Man9GlcNAc2 (9). Mean and standard error are shown for triplicate experiments. Positive glycan binding based on negative control antibody binding is shown as a filled bar. Open bars indicate negative binding values. Positivity thresholds for 7, 8a, 8b, and 9 are 0.2x10^4^, 0.15x10^4^, 0.15x10^4^, 0.2x10^4^ respectively. (TIF) [file ppat.1008165.s003.tif]
